# Hypercrosslinked core-shell polymer microspheres with amphipathic and amphoteric character for the solid-phase extraction of acidic and basic pharmaceuticals from environmental waters

**DOI:** 10.1007/s00216-026-06578-z

**Published:** 2026-05-28

**Authors:** Abril Trullàs, Judit Sánchez, Alan Corrigan, Francesc Borrull, Peter A. G. Cormack, Núria Fontanals, Rosa Maria Marcé

**Affiliations:** 1 Department of Analytical Chemistry and Organic Chemistry, Universitat Rovira I Virgili, Sescelades Campus, Marcel·lí Domingo 1, 43007 Tarragona, Spain; 2 WestCHEM, Department of Pure and Applied Chemistry, University of Strathclyde, Thomas Graham Building, 295 Cathedral Street, Glasgow, Scotland G1 1XL UK

**Keywords:** Core-shell format, Hypercrosslinked polymer, Amphoteric material, Mixed-mode ion-exchange, Solid-phase extraction, Environmental water samples

## Abstract

Solid-phase extraction (SPE) is used extensively in analytical chemistry for the preconcentration and fractionation of analytes prior to chemical analysis, with enhancements in sorbent efficiency and selectivity being important R&D topics. Although core-shell particles are increasingly used as alternatives to traditional high-performance liquid chromatography (HPLC) stationary phases, their value as SPE sorbents is under-explored. Herein, we describe the design, synthesis and exploitation of core-shell polymer microspheres, where the microspheres are small (3.7 µm) and uniform (*C*_v_ = 5.5%), and where the shells are thin (~0.5 µm, which improves mass transfer and reduces eddy diffusion) and have high specific surface area (>1300 m^2^/g) and amphoteric character. The latter feature means that these microspheres offer both anion-exchange and cation-exchange interactions, which makes the SPE of anions and cations possible. Following synthesis, an SPE protocol using these microspheres as sorbent was optimized and validated using liquid chromatography-tandem mass spectrometry (LC–MS/MS) for the simultaneous retention of acidic and basic pharmaceuticals present in complex water samples. The inclusion of a clean-up step reduced matrix interferences. The method developed achieved satisfactory apparent recoveries (23–75% for river water, 20–88% for effluent wastewater, and 19–56% for influent wastewater), low matrix effects, detection limits within 0.05–2 ng/L for most compounds, and good repeatability and reproducibility across all sample matrices. Application of the SPE method to real environmental samples confirmed the presence of several target pharmaceuticals at ng/L concentration levels, demonstrating the applicability of the hypercrosslinked core-shell sorbent.

**Graphical abstract:**

**Figure:**
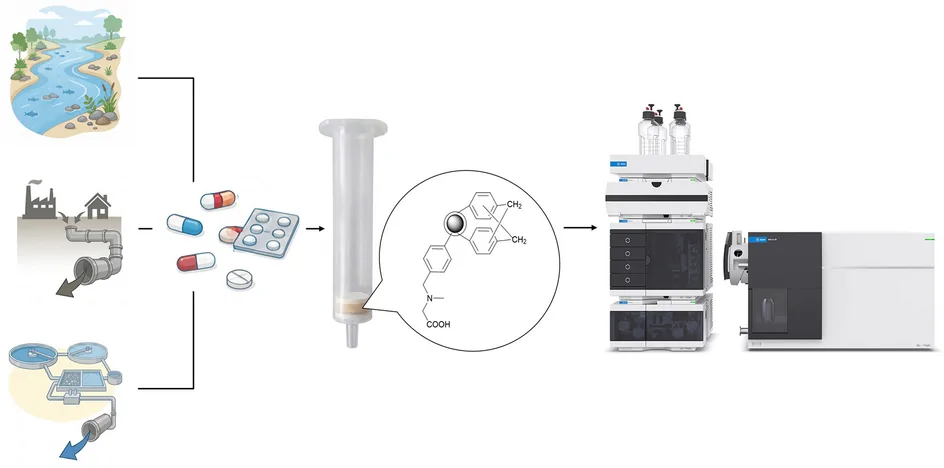

**Supplementary Information:**

The online version contains supplementary material available at https://doi.org/10.1007/s00216-026-06578-z.

## Introduction

Solid-phase extraction (SPE) is a widely used sorptive extraction technique for pollutants present in environmental water samples. This is because it is a simple, reliable and low-cost method for preconcentrating analytes. SPE also enables interferences to be separated from analytes, a feature that becomes especially important when complex matrices are being analyzed [1, 2]. In any given chemical separation scenario, the effectiveness of an SPE method depends on the chemical and physical nature of the sorptive material (sorbent) used. Most obviously, the chemical constitution of a sorbent dictates the types of non-covalent interactions that can be supported between the sorbent and the analytes, and thereby the extraction selectivity/mode of the sorbent; however, the physical format, porous morphology and stability of the sorbent are important practical considerations too. All of these variables influence the selectivity, utility and overall performance of SPE sorbents [3].

Most SPE sorbents are either silica-based or polymer-based, with many polymer-based sorbents having been developed in recent years to address practical limitations associated with traditional silica sorbents (e.g., limited pH stability, poor retention of highly polar compounds, difficulties with the elution of the analytes) [4–6]. More recent trends include the exploitation of metal–organic frameworks (MOFs) [7, 8] and covalent organic frameworks (COFs) [9] as sorbents, and the development and implementation of greener sorbents, such as cork, cellulose-based and biowaste-based materials [10].

While newer sorbents normally lead to improvements in performance, selectivity towards target analytes can still be a challenge. Molecularly imprinted polymers (MIPs) have been designed to address this need, since MIPs offer high levels of affinity and selectivity for the compounds used as templates in their production [11]. For instance, Khulu et al*.* [12] used a venlafaxine-imprinted polymer as an SPE sorbent and reported high selectivity towards selected pharmaceuticals in surface water samples, with extraction efficiencies between 43 and 69%. Similarly, mixed-mode ion-exchange materials enhance the selectivity for ionizable compounds. Typically, these materials exhibit multiple retention mechanisms, most commonly a combination of reversed-phase and ion-exchange interactions [3]; the silica or polymeric backbones in the sorbents confer the reversed-phase characteristics, whereas the immobilized functional groups are responsible for supporting the ion-exchange interactions.

Mixed-mode ion-exchange sorbents can be categorized into four main types, depending on whether the immobilized functional groups act as anion- or cation-exchangers, and whether the sorbents are categorized as strong or weak ion-exchangers [3, 13, 14]. Typically, strong cation-exchangers (SCX) contain sulfonic acid groups and strong anion-exchangers (SAX) possess quaternary ammonium groups, with both variants usually remaining permanently charged when in use irrespective of the pH. In contrast, weak cation-exchangers (WCX) normally contain carboxylic acid groups and weak anion-exchangers (WAX) contain primary, secondary and/or tertiary amine groups, with these variants being charged and capable of ion-exchange across certain pH ranges only, ranges that are determined by the pK_a_ values of the immobilized species [14, 15].

For SPE applications requiring simultaneous retention of anionic and cationic species, the use of cation- and anion-exchange SPE cartridges in tandem has been reported [16, 17]. However, a very interesting alternative option is the exploitation of amphoteric sorbents, a novel approach that we have pioneered [18]. Amphoteric compounds are compounds that are able to react both as a base and as an acid, therefore amphoteric SPE sorbents are sorbents bearing immobilized amphoteric groups that are capable of anion-exchange and cation-exchange [14, 19].

Polymer-based mixed-mode ion-exchange sorbents are normally based on crosslinked macroreticular polymers that are mechanically robust and offer good access to the analytes for the ion-exchange groups that are present throughout the crosslinked network. The installation of porosity into such sorbents through hypercrosslinking strategies rather than through the use of porogenic agents leads to sorbents with remarkably high specific surface areas (> 1000 m^2^/g) and correspondingly high sorption capacities, together with other benefits and unusual features including amphipathic character [20]. Such materials are highly tunable through the incorporation of ion-exchange moieties through polymerization or polymer-analogous reactions, and our group has reported extensively on the production of polymer microsphere variants where the microspheres are small in size and uniform and where all four main types of ion-exchange are realizable [21, 22]. Recently, we extended the chemistry to include polymer microspheres containing two ion-exchange groups simultaneously, using immobilized taurine to give WAX/SCX variants and immobilized quaternized sarcosine to give SAX/WCX variants [21]. When the immobilized species is non-quaternized sarcosine [18] then the variant has WAX/WCX properties and a very interesting finding was made—when such a sorbent was exploited in SPE, anions and cations could be captured simultaneously at intermediate pH.

Core-shell particles have been developed as alternatives to traditional high-performance liquid chromatography (HPLC) stationary phases, to improve chromatographic efficiency and separation power. In this format, the solid cores provide mechanical stability and analytes interact with the functional shells only. The format works well because the shells are normally relatively thin (< 1 µm is typical); this improves mass transfer and reduces eddy diffusion, which reduces band-broadening effects. While core-shell particles are gaining widespread acceptance for HPLC separations, and interesting new variants are being reported in the literature [22], their value as SPE sorbents is largely under-exploited. Our first foray into this core-shell area concerned the preparation of ion-exchange sorbents with SCX character [23], where sulfonic acids groups were introduced post-polymerization through an electrophilic aromatic substitution method. The development of core-shell materials having amphipathic and amphoteric character in the functional shells is particularly attractive and timely because it may be possible to take advantage of the very unusual solvent uptake properties of hypercrosslinked materials, the ultra-high specific surface areas arising through hypercrosslinking yields sorbents with high sorption capacities, and the fractionation properties of these structurally unusual sorbents can be switched reversibly between anion capture and cation capture or be used for the simultaneous capture of anions and cations. Such opportunities provided the motivation for the work described in the present paper, and yielded interesting synthetic challenges, such as the need to avoid exhaustive hypercrosslinking in order to allow for the introduction of amino acid moieties through nucleophilic aliphatic substitution reactions on polymer-bound chloromethyl groups.

## Experimental

### Standards and reagents

For the synthesis of the core-shell polymer microspheres, the monomers divinylbenzene-55 (DVB-55) (55%, technical grade), divinylbenzene-80 (DVB-80) (80%, technical grade) and 4-vinylbenzyl chloride (VBC) (≥ 90%) were purchased from Sigma-Aldrich (Saint Louis, MO, USA) and purified prior to use by percolation through a short column of neutral aluminum oxide. The free radical initiator 2,2′-azo*bis*isobutyronitrile (AIBN) (98%) was used as received from Sigma-Aldrich. All other reagents and solvents were used as received from commercial suppliers: acetonitrile (ACN) (99.8%, HPLC grade), methanol (MeOH) (≥ 99.5%, analytical specification), sodium hydrogen carbonate and potassium hydroxide from VWR chemicals (Portland, OR, USA); FeCl_3_, 1,2-dichloroethane (DCE) (anhydrous, 99.8%) and sarcosine ethyl ester hydrochloride (≥ 99%) from Sigma-Aldrich; toluene (≥ 99.8%, analytical grade) from Fischer Chemicals; tetra-*n*-butylammonium bromide (TBAB) (> 98%) from Alfa Aesar.

Thirteen analytes were selected for the SPE study: seven acidic pharmaceuticals (bezafibrate [BEZ], diclofenac [DICLO], flurbiprofen [FLB], fenoprofen [FEN], naproxen [NPX], valsartan [VAL], and the clofibrate metabolite clofibric acid [CLO]), and six basic pharmaceuticals (propranolol [PRO], atenolol [ATE], metoprolol [MTO], trimethoprim [TRI], ranitidine [RAN], and venlafaxine [VEN]). All thirteen compounds were acquired from Sigma-Aldrich (St. Louis, USA) in a purity > 96%. Their chemical structures, pK_a_ values and other relevant data are presented in Table S1.

Stock solutions of individual standards (1000 mg/L) were prepared in MeOH and stored at −20 °C. Working solutions containing a mixture of all analytes were prepared weekly in ultrapure water and MeOH (90/10, v/v) and stored at 4 °C in the dark. Ultrapure water was provided by a water purification system (Millipore, Burlington, United States), while HPLC-grade MeOH and acetonitrile (ACN) were purchased from J. T. Baker (Deventer, The Netherlands). LC–MS grade ACN and water were purchased from Carlo Erba (Val de Reuil, France). Formic acid (> 98%) and HCl (> 37%, reagent grade) were used to adjust pH of the mobile phase and were acquired from Sigma-Aldrich. Ammonium hydroxide 28% was purchased from Thermo Scientific.

### Synthesis of core-shell polymer microspheres

The core-shell polymer microspheres were synthesized via a five-step synthetic protocol: (i) precipitation polymerization (PP) of DVB-55 to give polyDVB-55 seed particles; (ii) shelling of the polyDVB-55 seed particles by the PP of DVB-80 and VBC in the presence of the seed particles to give polyDVB-55@poly(DVB-80-*co*-VBC); (iii) hypercrosslinking of the poly(DVB-80-*co*-VBC) shells; (iv) chemical immobilization of sarcosine ethyl ester through a polymer-analogous reaction on pendent chloromethyl groups; (v) hydrolysis of the ethyl ester groups in the immobilized sarcosine ethyl ester residues. Synthetic details are reported in the Electronic Supplementary Material (ESM).

### Characterization of core-shell polymer microspheres

ATR-FTIR spectra were acquired using a Thermo Scientific Nicolet iS5 FTIR spectrometer. Samples were scanned 16 times each, over the range 400–4000 cm^−1^. Spectra were recorded and analyzed using Omnic Series Software from Thermo Scientific. Scanning electron microscopy (SEM) was conducted using a Cambridge Stereoscan 90 Scanning Electron Microscope; prior to imaging, samples were sputter-coated with gold using a Polaron SC500A Sputter Coater. Mean particle diameters (n = 100) were determined using ImageJ (v1.54, National Institutes of Health) open-source image processing and analysis software. Nitrogen sorption analysis was carried out using a Micromeritics ASAP 2020 Porosimeter. The characterization data for all of the polymers synthesized is reported in the ESM.

### SPE protocol

A 6 mL SPE cartridge (Symta, Madrid, Spain) was packed with 150 mg of sorbent between a 10 µm polyethylene (PE) frit (Symta) at the bottom, a 2 µm stainless steel frit (Sigma Aldrich) above the PE frit, and a second 10 µm PE frit placed on top of the sorbent.

The extraction protocol started with sorbent conditioning using 5 mL of MeOH, followed by 5 mL of ultrapure water adjusted to pH 6. Subsequently, a sample volume of 100 mL, also adjusted to pH 6, was loaded into the cartridge. A clean-up step was then performed using 0.5 mL of MeOH, followed by the elution stage with 5 mL of 5% NH_4_OH in MeOH. The eluate was evaporated to dryness using a miVac Duo centrifugal evaporator (Genevac, Ipswich, UK) and reconstituted with 1 mL of water/MeOH (95/5, v/v). All reconstituted extracts were filtered through 0.22 µm polytetrafluoroethylene (PTFE) filters (Scharlab), prior to injection.

### Chromatographic conditions

SPE procedure parameters were optimized with an Agilent 1200 UHPLC instrument equipped with an automatic injector, an autosampler, an oven, a binary pump, a degasser, and a diode array detector (DAD). The column used for the chromatographic separation was a Luna Omega 5 μm Polar C_18_ 100 Å (150 × 3 mm, 5 μm particle size) with a precolumn (4 mm × 3 mm, 5 μm), both supplied by Phenomenex (Torrance, CA, USA). The temperature of the oven was fixed at 30 °C, the injection volume was 20 μL, and the flow rate was 0.4 mL/min. A binary mobile phase consisting of ultrapure water (A) and ACN (B), both adjusted to pH 3 with HCl, in a gradient elution program was used to perform the separation. The program started at 5% of B, progressively increasing to 40% within 10 min, then to 45% in 4 min, and finally reaching 100% in 5 min. The % of B was held at 100% for 2 min, and then returned to the initial conditions within 2 min. These conditions were held for 5 min for the stabilization of the column. All compounds were measured at 210 nm.

Once the key parameters within the SPE protocol were optimized, an LC–MS/MS instrument was used to validate the method and analyze environmental samples. Specifically, an Agilent 1200 instrument equipped with an autosampler and a quaternary pump coupled to a 6460 QqQ mass spectrometer (MS/MS) (Agilent Technologies, Santa Clara, CA, USA) was used. The chromatographic conditions described above were used, except that the injection volume was reduced to 5 μL, and 0.1% HCOOH replaced HCl in both mobile phases (A and B). Regarding the MS/MS conditions, electrospray ionization (ESI), working in both negative and positive modes, was selected as the ionization interface in the MS/MS for the acidic and basic compounds, respectively. The detection parameters for the acquisition of all the compounds were as follows: a nebulizer pressure of 50 psi, a nitrogen flow rate of 12 L/min, a source gas temperature of 320 °C, a capillary voltage of 3000 V for positive mode and 3500 V for the negative mode, a fragmentor voltage between 70 and 100 V, and collision energies between 2 and 25 eV. Data acquisition was performed in multiple reaction monitoring (MRM) mode, with the selection of a precursor ion and two product ions for each analyte. The most abundant transition was selected as quantifier and the other transitions and their corresponding ion ratios were used to qualify in the MRM. Table S1 presents the corresponding transitions, collision energies and fragmentor voltages of all analytes under study.

### Validation parameters

After optimizing the SPE protocol, the whole method was validated using a more sensitive and selective instrument (LC–MS/MS). Prior to method validation, instrumental performance was assessed in terms of linearity and instrumental limits of quantification (LOQ) and limits of detection (LOD), which were obtained as the concentration that accomplishes the signal-to-noise ratio of 10 and 3, respectively. Instrumental calibration curves showed good linearity (R^2^ > 0.997) across a concentration range from LOQ to 500 µg/L. Instrumental LOQs were defined as the lowest concentration of the calibration curve, which was 0.1 µg/L for most basic analytes, except TRI for which the LOQ was 0.25 µg/L. The acidic compounds exhibited higher LOQs: 0.25 µg/L for BEZ and VAL; 0.5 µg/L for CLO, NPX, and DICLO; 5 µg/L for FLB; and 10 µg/L for FEN. Furthermore, instrumental LODs, defined as the concentration corresponding to a signal-to-noise ratio > 3, were as low as 0.02 µg/L for all basic compounds; 0.1 µg/L for CLO, BEZ, VAL, and DICLO; 0.25 µg/L for NPX; 1 µg/L for FLB; and 5 µg/L for FEN. The higher LOQs and LODs for FEN and FLB were attributed to poor ionization efficiency.

Given the use of LC–MS/MS, matrix effect (%ME) was a parameter necessary to assess in this method, as well as apparent recovery (%R_app_), and precision. %R_app_ values were determined experimentally by spiking samples with a mixture of analytes at different concentration levels: 0.2 and 2 µg/L for river water, 2 µg/L for effluent wastewater, and 5 µg/L for influent wastewater samples. They were calculated by subtracting the concentration measured in the corresponding blank from the concentration measured in the spiked sample, and dividing this difference by the theoretical spike concentration.

%ME values were evaluated by spiking a blank sample after the SPE protocol. The calculation was performed using the following expression: %ME = [(C_exp_/C_theo_) × 100] − 100, where *C*_*exp*_ is the difference between the concentration obtained from the calibration curve and that measured in the non-spiked blank extract, and *C*_*theo*_ is the theoretical concentration. Positive values denote signal enhancement whereas negative values indicate signal suppression.

Method accuracy was assessed in terms of relative recoveries (%R_rel_), which were calculated as the ratio between the concentration after applying %R_app_ and the theoretical concentration. It was determined at different concentration levels: 0.2 and 2 µg/L for river water, 2 µg/L for effluent, and 5 µg/L for influent wastewater samples. Method precision was evaluated in terms of repeatability, including intra-day and inter-day. Intra-day precision was assessed by quintuplicate extractions (n = 5) performed within a single day using one single extraction device, and inter-day precision was assessed over five consecutive days (n = 5). All precision values were expressed as relative standard deviation (% RSD) for the same concentration levels mentioned in apparent recoveries.

Finally, method detection limits (MDLs) and method quantification limits (MQLs) were estimated from LODs and LOQs, and applying the preconcentration factor and the apparent recoveries to the corresponding instrumental limits.

## Results and discussion

### Synthesis and characterization of core-shell polymer microspheres

Core-shell polymer microspheres with amphipathic and amphoteric character were synthesized through two consecutive precipitation polymerizations (PP), followed by a hypercrosslinking reaction, followed by two polymer-analogous reactions (Figure S1). The amphipathic character of the shells arises from the fact that they are hypercrosslinked (amphipathic character is an intrinsic property of hypercrosslinked polymers), whereas the amphoteric character is derived from the sarcosine (*N*-methylglycine) residues that are introduced into the shells through the polymer-analogous reactions. PP was used as the preferred method of synthesis since this method delivers clean, high-quality particles in the micron-size range in one single preparative step and allows for the production of core-shell formats; non-porous polyDVB-55 microspheres from the first PP were used as seed particles in a second PP where the co-monomers were DVB-80 and VBC, to give non-porous core-shell microspheres. Subsequently, VBC residues in the shells provided a source of internal electrophiles in a hypercrosslinking reaction, a very efficient Friedel–Crafts alkylation reaction that was conducted in a solvent-swollen state and for a restricted time period (10 min) to install porosity yet leave a portion of functional group handles (chloromethyl groups) available for the subsequent polymer-analogous reactions. The latter installed the sarcosine residues. The core-shell microspheres synthesized in this manner were very uniform (Figure S2e).

The core-shell polymer microspheres produced and used for the SPE study had a mean particle diameter of 3.7 µm; they were quasi-monodisperse (C_v_ = 5.5%) and had a shell thickness around 0.5 µm. Furthermore, the core-shell microspheres had a Langmuir specific surface area of 820 m^2^/g and a mean pore diameter of 3.2 nm. Given that the polyDVB-55 cores are non-porous and will not contribute to nitrogen uptake in the nitrogen sorption analysis, a very conservative estimate of the specific surface area of the shells is > 1300 m^2^/g. Such materials are attractive candidates as SPE sorbents.

### Optimization of SPE parameters

The core-shell polymer microspheres were evaluated as sorbent for the SPE of thirteen acidic and basic pharmaceuticals (see Table S1 for chemical structures and pK_a_ values). To enhance the extraction performance, key parameters of the SPE protocol were investigated and optimized in triplicate, including sample pH and volume, the MeOH clean-up volume, and the elution conditions. Extraction recoveries (% R)—calculated as the ratio between the concentration obtained after SPE and the theoretical spiked concentration in ultrapure water (0.1 mg/L)—were used to determine the optimal conditions.

The initial SPE conditions used were guided by a report by Nadal et al*.* [18] in which a fully porous (i.e., not a core-shell format) sarcosine-containing sorbent was under investigation. In outline, 150 mg of packed sorbent was conditioned with 5 mL of MeOH, followed by 5 mL of ultrapure water (pH 6). Extraction was conducted using 10 mL of ultrapure water spiked with the target analytes at 0.1 mg/L, adjusted to a range of different pH values. A clean-up step was then performed with 1 mL of MeOH. Then, two consecutive elution steps were carried out with 5 mL of 5% NH₄OH in MeOH in each step. The eluate was evaporated to dryness under vacuum and reconstituted in 1 mL of ultrapure water/MeOH (95/5, v/v) prior to LC-DAD analysis.

#### Sample pH

**Fig. 1:**
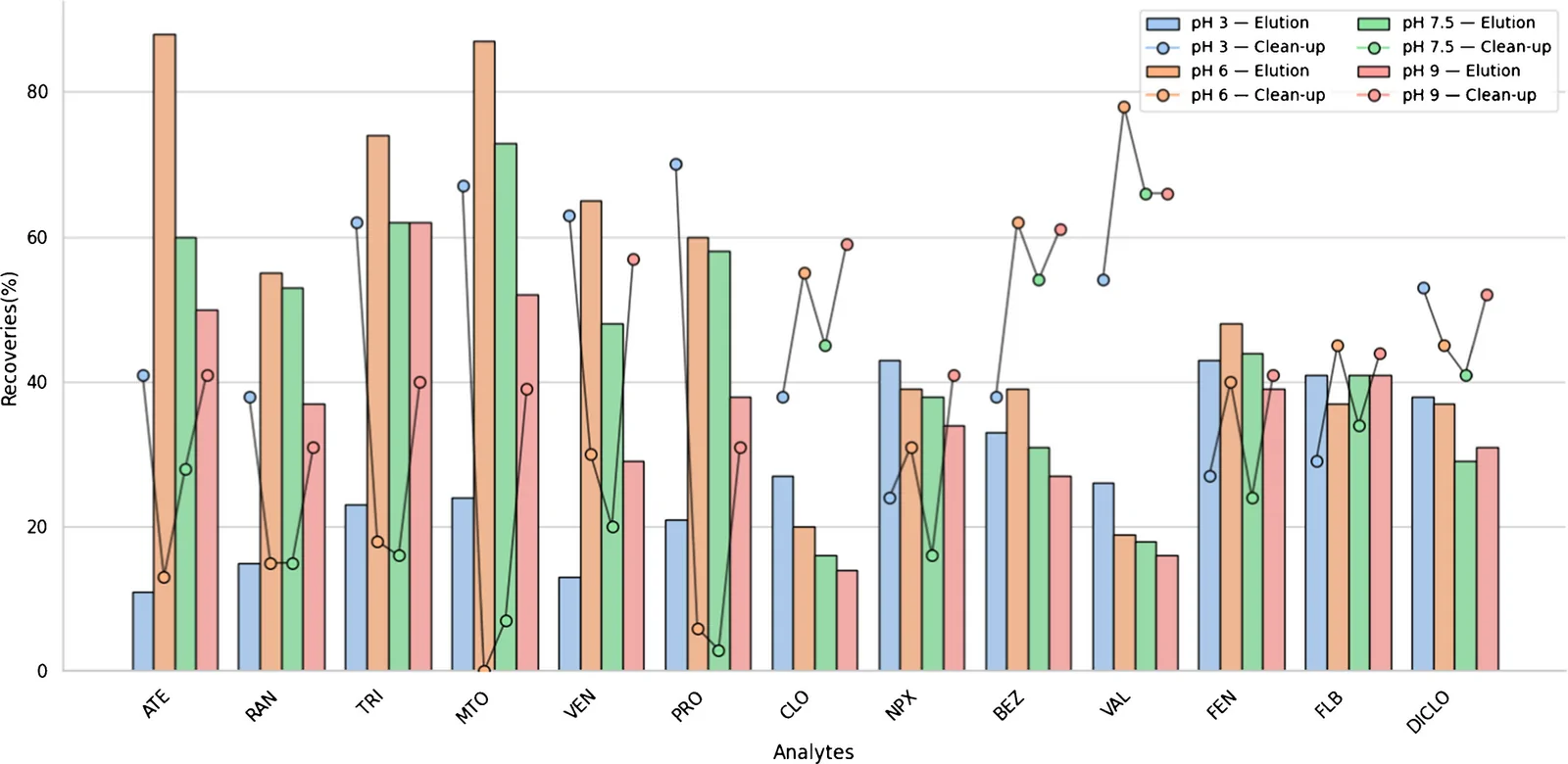
Elution recoveries and clean-up losses (%) during sample pH assessment

Sample pH is a crucial parameter when evaluating materials with mixed-mode ion-exchange properties, as only specific pH ranges will ensure effective ionic interactions between the ion-exchange sites in the sorbent and the analytes. In the present study, the sorbent has two ion-exchange motifs (WAX and WCX) arising from tertiary amine groups and carboxylic acid groups, respectively. WAX interactions will occur only when the polymer-bound amine groups are positively charged (at pH values lower than ~ 10) and the acidic analytes are deprotonated (at pH values higher than their pK_a_, > 4.5, see Table S1). On the other hand, WCX interactions will be possible only when the polymer-bound carboxylic acid groups are negatively charged (at pH values above ~ 2) and the basic analytes are protonated at values below their pK_a_ (< 7, see Table S1). Therefore, mixed-mode ionic interactions are only expected for this sorbent at intermediate pH values. For this reason, and as stated in “Optimization of SPE parameters”, the initial conditions included the loading of the sample at pH 6. Figure 1 shows the influence of pH on analyte retention, expressed as colored bars representing elution recoveries and colored dots depicting clean-up losses at each pH. The initial test at pH 6 resulted in satisfactory recoveries for basic compounds (between 55 and 88%), whereas acidic compounds provided lower recoveries, between 10 and 48%. Furthermore, during the clean-up step the basic analytes were less prone to being lost than the acidic analytes, suggesting that the WCX interactions were stronger than the WAX interactions under these conditions, and that non-specific interactions contributed to retention as MeOH was able to disrupt them.

Thereafter, to provide a more detailed insight into the dependency of retention on the loading pH, three additional pH values for the loading solution were tested (3, 7.5 and 9) while keeping the other SPE parameters constant.

As Fig. 1 illustrates, at pH 3 the tertiary amine groups within the sorbent are protonated and capable of retaining anions. However, because the working pH is below the pK_a_ values of the acidic analytes (3.2–4.7) then these compounds remain uncharged at pH 3 thereby limiting ionic interactions and favoring non-selective interactions. The basic analytes, which are protonated under these conditions, were also poorly retained since the carboxylic acid groups within the sorbent are undissociated at pH 3. Nevertheless, the acidic and basic analytes were partially retained through weak, non-selective interactions. The uncharged acidic compounds showed a greater tendency to interact through non-specific hydrophobic mechanisms with the hypercrosslinked polymer framework, which explains their higher recoveries (26–43%). In contrast, the ionization state of the basic analytes disfavors hydrophobic interactions with the polymeric backbone, resulting in weaker retention and lower recoveries (11–24%). Moreover, during the MeOH clean-up step, the basic analytes were more easily washed away, confirming the disfavored (weaker) non-selective interactions of these compounds. For instance, 60% of the basic analytes TRI and VEN were lost in the washing step compared to around 30% for the acidic compounds FEN and FLB, as presented in Fig. 1.

At pH 9, the acidic analytes are ionized, but the tertiary amine groups within the sorbent are not fully protonated, reducing anion-exchange retention. In contrast, the basic analytes are partially ionized at pH 9, and the carboxylic acid groups within the sorbent are fully ionized, promoting WCX interactions. Consequently, the basic compounds had higher recoveries (29–62%) than the acidic compounds (14–41%) at pH 9, verifying the predominance of WCX character over WAX character at high pH.

At pH values close to neutral (6 and 7.5), the amine groups and carboxylic acid groups within the sorbent are charged, and this is also the case for the acidic and basic analytes in solution. Therefore, cation-exchange and anion-exchange are both feasible at these intermediate pH values. Compared to pH 6, the recoveries of the acidic and basic analytes at pH 7.5 were a little bit lower, therefore pH 6 was considered to provide optimal retention. Regarding the clean-up step, the level of losses of the basic compounds at pH 6 and pH 7.5 was similar, with the exception of ATE for which approximately 30% was washed away at pH 7.5 compared to 13% at pH 6. In contrast, VEN showed the opposite behavior, with a higher percentage lost at pH 6. As can be observed in Fig. 1, acidic analytes were lost to a greater extent at the intermediate pH values (6 and 7.5) than at pH 3, with losses ranging from 16 to 78%. Consequently, lower recoveries were obtained, suggesting that acidic analytes were retained mainly through non-selective interactions. Overall, even though the acidic analytes were partially washed away, the recoveries of the basic compounds were higher at pH 6 than at pH 7.5, therefore pH 6 was selected as the optimal condition. These findings are in agreement with data reported by Nadal et al*.* [18], whose study focused on the simultaneous extraction of acidic and basic compounds using an SPE sorbent with amphoteric character. In their study, they observed the predominance of WAX character at lower pH values, WCX dominance at higher pH values, and both WAX and WCX character at intermediate values, and so selected pH 6 as the optimal conditions for the simultaneous extraction of acidic, basic, and amphoteric analytes.

#### Elution conditions

Up until this point in the study, a solution of 5% NH₄OH in MeOH had been used as the elution solvent. This basic additive neutralizes cationic species, thereby deprotonating the amino groups in the sorbent and switching off the WAX mechanism, as well as neutralizing basic analytes that are bound to the sorbent through WCX interactions. 5% CH_3_COOH in MeOH was tested as an alternative elution solvent. This acidic additive protonates anionic species, thereby protonating the carboxylic groups in the sorbent and the acidic analytes that are bound to the sorbent through the WAX mechanism. To ensure complete elution of all analytes, a second elution step was implemented: for the acidic elution 5% CH_3_COOH in MeOH, and 5% NH₄OH in MeOH in the case of basic elution.

**Fig. 2:**
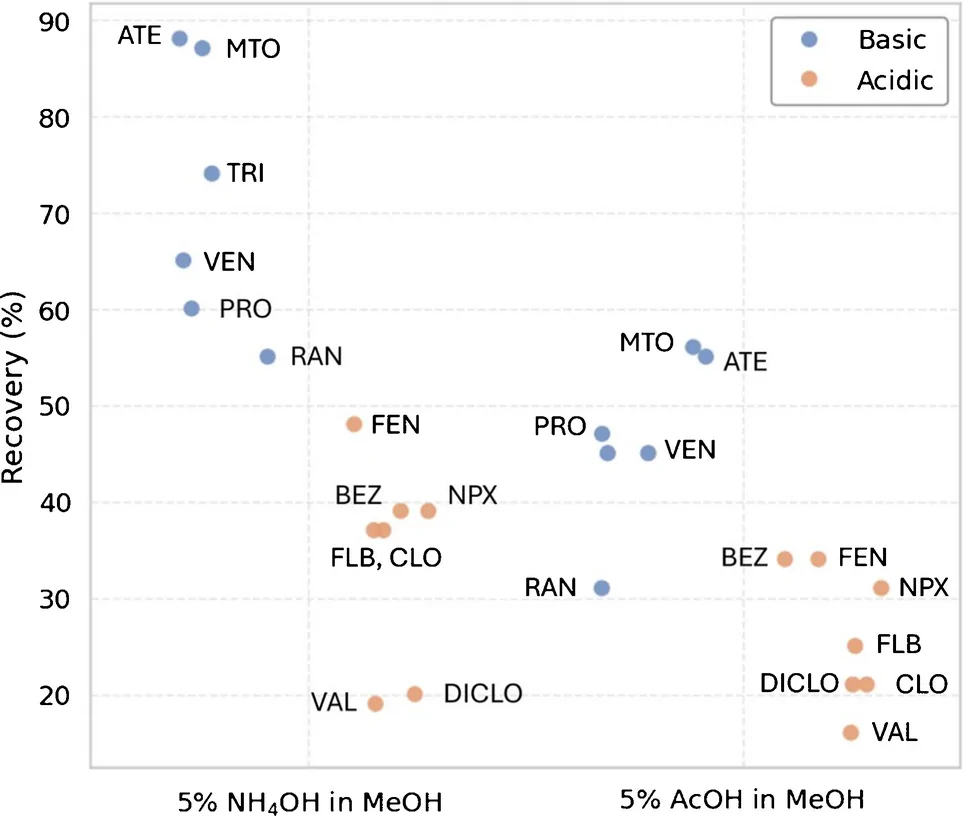
Scatter plot of the recoveries of the basic (blue) and acidic (orange) analytes as a function of the elution solvent used

Figure 2 displays a scatter plot of the recoveries of the acidic analytes (orange) and the basic analytes (blue) as a function of the elution solvent used. As shown in the plot, all analytes were desorbed efficiently by both elution solvents. Under the basic elution conditions, recoveries for the basic analytes ranged between 55 and 88%, and between 19 and 48% for the acidic analytes. In contrast, when using the acidic elution conditions, the recoveries ranged from 31 to 56% for the basic analytes and from 16 to 34% for the acidic analytes. The conclusion here is that basic elution provided higher recoveries than acidic elution for most compounds. Figure 2 also shows a minor limitation of the novel sorbent, which is the weaker performance of the WAX mechanism, as the acidic compounds are observed to be weakly retained in terms of lower recoveries compared to basic analytes, under both elution solvents.

The low recoveries from the second elution step (0–9%) indicated that all analytes were mostly desorbed during the first elution step, and that the second elution was not necessary. Consequently, one 5 mL portion of 5% NH₄OH in MeOH was selected as the optimal elution solvent.

Based on previous experience from within our research group [19, 24], 5% NH_4_OH in MeOH was selected as the percentage of additive present in the elution solvent. For instance, Gilart et al*.* [24] assessed three commercial sorbents (Oasis HLB, Oasis WAX and Oasis SAX) for the extraction of drugs from wastewater samples and compared the elution of acidic analytes using 2% and 5% of NH_4_OH in MeOH. It was reported that 5% of NH_4_OH in MeOH yielded higher extraction recoveries for Oasis WAX. Subsequently, Moral et al*.* [19] compared 5% and 10% NH_4_OH in MeOH for the elution of both acidic and basic analytes from SiO_2_-C-SAX/SCX and SiO_2_-SAX/SCX sorbents, and observed no significant differences in recoveries (85–105%). All in all, these results suggest that 5% NH₄OH in MeOH is generally effective across polymeric sorbents having variable structures, supporting its use in the present method without further optimization.

#### Methanol-based clean-up volume

Sorptive extraction techniques often employ clean-up steps to remove interferences from complex samples and to enhance selectivity of the method. In this study, the clean-up with MeOH disrupts non-specific (non-ionic) interactions between organic matrix components and the sorbent, leading to the elimination of undesired compounds from the sorbent. As a result, during the elution step non-ionic matrix-derived compounds are absent, allowing the selective extraction of ionizable target analytes.

In the previous tests, 1 mL of MeOH was used as the clean-up solvent, and to evaluate whether analyte losses during the washing step could be minimized, a lower volume was tested (0.5 mL). Upon reducing the MeOH volume to 0.5 mL, it was observed that the losses of acidic analytes were reduced: the recovery of acidic analytes ranged between 38 and 56%, compared to 19–48% when the volume of MeOH was 1 mL. In contrast, the recoveries of the basic analytes remained constant (38–90%) regardless of the volume of clean-up solvent used.

A similar observation was reported by Moral et al*.* [19], who investigated the effect of different MeOH wash volumes (2 and 5 mL) for an SPE protocol employing a zwitterionic silica-based sorbent. For both wash volumes, the sorbent exhibited comparable performance, with recoveries above 80% for basic compounds and below 10% for acidic compounds. These results confirmed that acidic analytes were retained predominantly through hydrophobic interactions, as they were removed easily from the sorbent during the washing with MeOH. Conversely, basic analytes were retained through ionic-exchange interactions and were not eluted during the clean-up step. Consequently, they selected a clean-up solvent volume of 5 mL to enhance the selectivity towards basic compounds. This last conclusion by Moral et al*.* differs from the present study, in which the solvent volume was reduced (down to 0.5 mL) to minimize the loss of basic analytes during this step, but also to reduce the overall solvent waste.

#### Sample volume

**Fig. 3:**
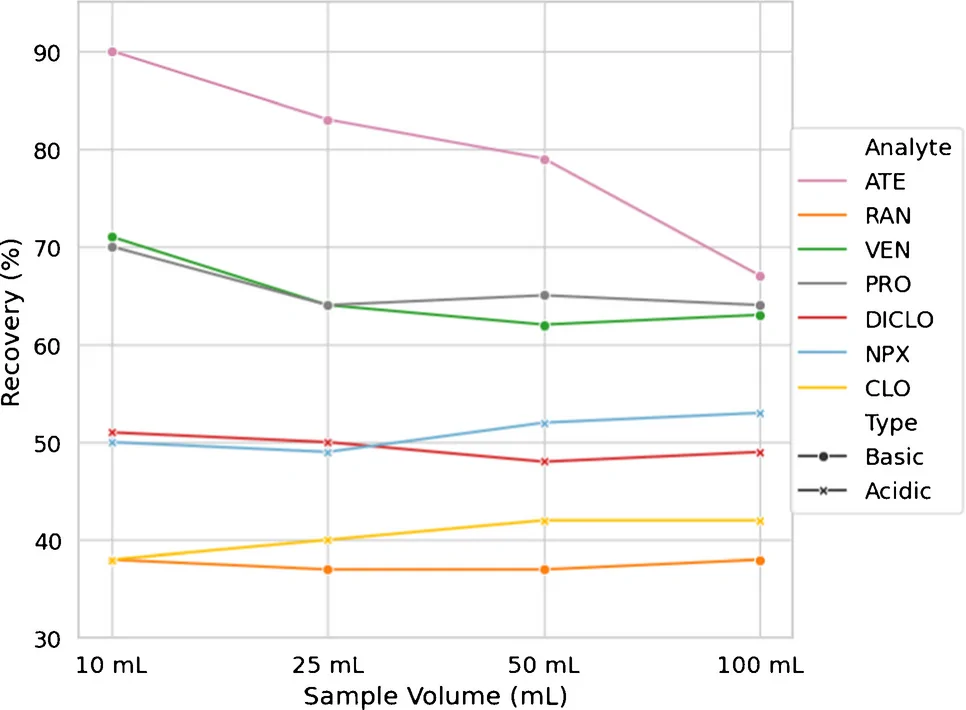
Dependence of recoveries (%) on sample volume for seven analytes

To improve the method’s sensitivity and capacity, the effect of increasing the sample volume was studied, starting from 10 mL and increasing to 25, 50, and 100 mL. The corresponding results for representative analytes are presented in Fig. 3. Additionally, Figure S12 presents the recoveries of the remainder of analytes under study. It was found that the recoveries of acidic analytes did not show any significant decrease as the sample volume increased, whereas the recoveries of some basic analytes did decrease. For this reason, sample volumes greater than 100 mL were not tested. The recovery of ATE decreased steadily with increasing sample volume, from 90% at 10 mL to 67% at 100 mL. VEN and PRO also experienced a slight decrease, from approximately 70% at 10 mL to 64% at 25 mL. In both cases, the extraction efficiencies remained constant from 25 to 100 mL. The recoveries of the other basic analytes remained relatively stable, showing only a minor decrease of less than 5%.

Nadal et al*.* [18] also optimized the sample loading during the evaluation of a hypercrosslinked polymer material having WAX and WCX character for the SPE of basic and acidic pharmaceuticals from environmental water samples. They evaluated sample volumes ranging from 25 to 250 mL and obtained recoveries above 78% for most compounds when the sample volume was 250 mL, although weakly retained analytes, such as acetaminophen (ACE), exhibited low recoveries (3%). Nonetheless, due to the complexity of the environmental samples, a loading volume of 100 mL was selected as the optimal. Similarly, Klančar et al*.* [25] investigated four loading volumes (100, 200, 500, and 1000 mL). Unsurprisingly, they reported a direct correlation between sample volume and loading time, reaching up to 3 h for 1000 mL; however, increasing the sample volume also led to cartridge clogging, causing sample loss and random variability. In addition, polar analytes showed significant decreases in recoveries as the sample volume was increased. Consequently, a loading volume of 200 mL was selected as optimal, since this provided satisfactory extraction efficiency and acceptable loading times. Consistent with the literature, increasing the sample volume improved the method sensitivity but led to a decrease in recoveries for weakly retained basic analytes, indicating sorbent capacity limitations due to the low breakthrough volume. Accordingly, a sample volume of 100 mL was selected as optimal for the present work, as this provided an acceptable balance between loading time, preconcentration and extraction efficiency.

### Validation of the method

The optimized method was validated for river, effluent and influent wastewater samples using an LC–MS/MS instrument. The validation parameters included apparent recovery (%R_app_) at different concentration levels, matrix effect (%ME), method accuracy (relative recoveries, %R_rel_), linear range, method detection limits (MDLs), method quantification limits (MQLs), and precision in terms of repeatability and reproducibility. It should be noted that due to the higher complexity of influent wastewater, and to attain similar recoveries to less complex matrices, the loading volume for these samples was reduced from 100 to 50 mL, whereas the optimized loading volume of 100 mL was maintained for river water samples and effluent wastewater samples.

**Fig. 4:**
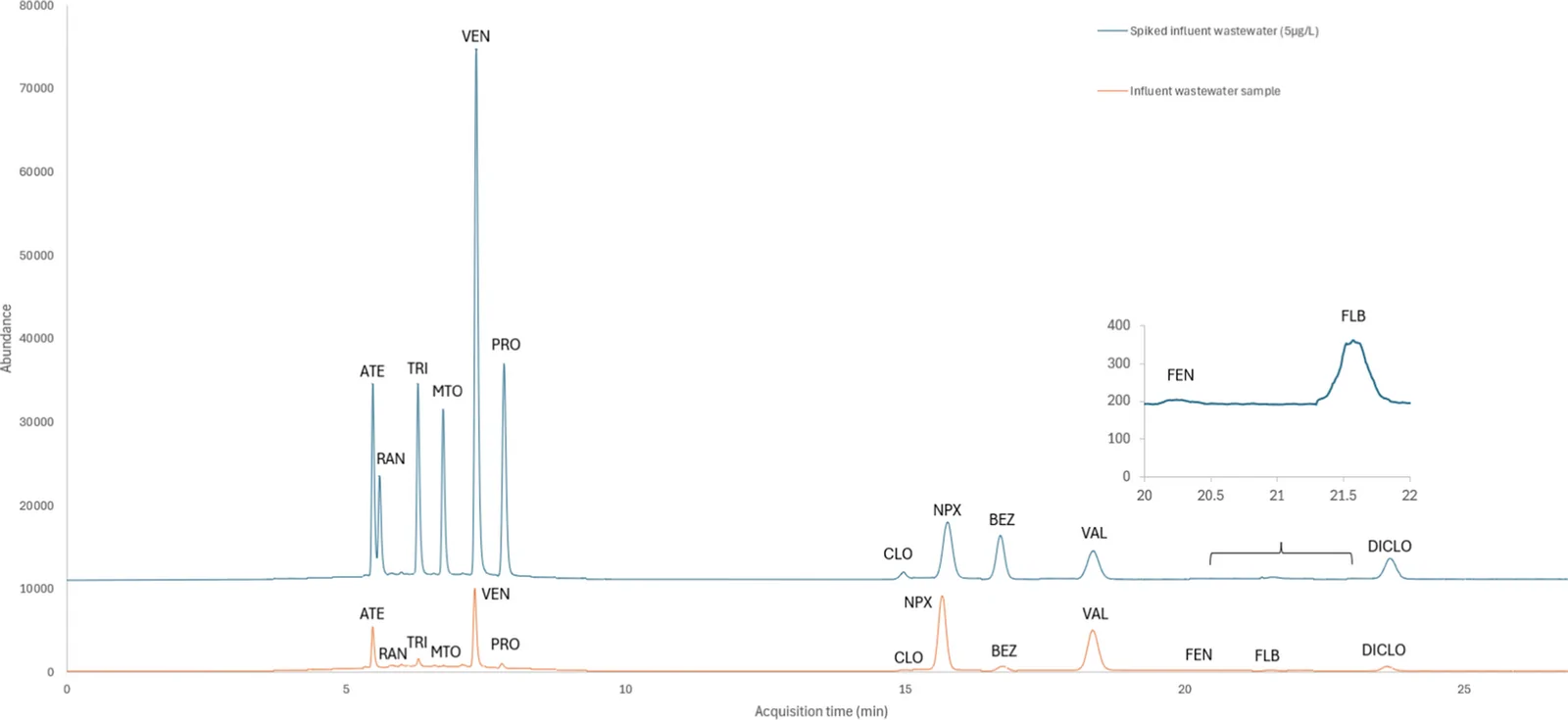
Total ion chromatogram of an influent wastewater sample with (blue) and without (orange) spiking at 5 µg/L concentration level, analysed using the optimized SPE-LC–MS/MS method

The %R_app_ values were evaluated by spiking the three water sample types at different concentration levels: 0.2 and 2 µg/L for river water, 2 µg/L for effluent, and 5 µg/L for influent wastewater samples. It must be noted that the effluent and influent wastewater samples used for the validation exhibited the natural occurrence of high concentrations of some analytes, and for this reason lower spiking levels were not evaluated. Figure 4 shows the high concentration of NPX and VAL in non-spiked influent wastewater compared to the spiked sample. Specifically, the concentrations of VAL and NPX in the influent wastewater samples exceeded the spiked levels; thus, %R_app_ could not be calculated. As shown in Table 1, good apparent recoveries were obtained, with values higher than 30% for most compounds except for PRO in river water, FEN in effluent wastewater, FLB in both effluent and influent wastewater, and RAN in influent wastewater samples, with values ranging from 19 to 30%. In general, a decreasing recovery trend was observed with increasing sample matrix complexity. For example, DICLO recoveries decreased from 62 to 68% for river water to 56% for effluent wastewater, and then to 48% for influent wastewater. In contrast, CLO and FEN exhibited higher recoveries for influent wastewater than for effluent wastewater samples. This behavior can be explained by the reduction of sample volume applied to influent wastewater (50 mL), which likely mitigated matrix interferences. Similar behavior was observed by Moral et al*.* [23], who reported higher %R_app_ values for influent wastewater samples than effluent wastewater samples (39–59% vs. 56–69%) for ATE, TRI and MTO, also as consequence of reduced loading volumes, when using a SCX core-shell sorbent. Nadal et al*.* [21] reported recovery values ranging from 18 to 93% for river water and 24–105% for effluent wastewater samples, using a SAX and WCX-functionalized hypercrosslinked polymeric sorbent for the extraction of acidic and basic pharmaceuticals. Moreover, Gilart et al*.* [24] compared the analytical performance of several commercially available sorbents. The recovery values for effluent wastewater samples ranged between 89 and 103% for common acidic and basic analytes using Oasis HLB, ranging between 98 and 105% for acidic compounds using Oasis WAX, and ranging between 63 and 85% for acidic compounds using a commercial MIP for non-steroidal anti-inflammatory drugs (Affinilute MIP–NSAIDs). Furthermore, Deeb et al*.* [26] also evaluated analytical performance on tandem commercial sorbents to achieve simultaneous extraction of basic and acidic compounds within surface and wastewater samples. In their study, the use of tandem Oasis (MAX + MCX) enabled the effective extraction of common analytes like DICLO and MTO with recoveries ranging between 93 and 101% and between 92 and 110%, respectively.

**Table 1:** Apparent recoveries (R_app_ (%)) at different concentration levels and matrix effects (%ME) for river water and effluent and influent wastewater samples

		Ebre river water			Effluent wastewater		Influent wastewater	
		R_app_ (%)^b^	R_app_ (%)^c^	ME (%)^b^	R_app_ (%)^c^	ME (%)^c^	R_app_ (%)^d^	ME (%)^d^
Basic	ATE	58	74	−5	69	−10	49	−20
	RAN	37	35	−4	30	−29	23	−35
	TRI	37	69	−21	56	−22	42	−30
	MTO	45	66	−19	61	−21	51	−27
	VEN	36	67	+ 5	51	−14	40	−19
	PRO	23	49	−18	54	+ 5	50	−9
Acidic	CLO	31	47	+ 8	40	−6	56	+ 3
	NPX	75	89	+ 15	60	+ 8	-^a^	-^a^
	BEZ	41	60	−17	59	−20	46	−26
	VAL	47	44	−10	88	+ 15	-^a^	-^a^
	FEN	35	27	+ 6	20	−30	17	+ 2
	FLB	33	36	−3	28	−9	17	−11
	DICLO	62	68	+ 1	56	−1	48	−5

^a^Analyte concentration higher than the spiked level

^b^Spiked at low level (0.2 µg/L)

^c^Spiked at high level (2 µg/L)

^d^Spiked at 5 µg/L

%ME values corresponding to the evaluated concentration levels are reported in Table 1. Overall, the values were low (within ± 30%) and mostly showed signal suppression. The only exception was RAN in influent wastewater, which presented a %ME of −35%, a value that is still considered low. %ME observed for effluent and influent wastewater samples was comparable, most likely due to the reduced loading volume used for influent wastewaters. In contrast, Moral et al*.* [14] reported considerably higher matrix effects for silica-based mixed-mode zwitterionic ion-exchange sorbents, with %ME values ranging from −46 to + 14% for river water samples, −49 to −3% for effluent wastewater samples, and −84 to −16% for influent wastewater samples. These elevated values were attributed to the omission of a washing step within their SPE protocol.

Satisfactory accuracy results were obtained with %R_rel_ values ranging from 85 to 112% (for river), from 99 to 101% (for effluent wastewater), and from 99 to 103% (influent wastewater). Relative recoveries were not determined for NPX and VAL for the influent wastewater, as their %R_app_ could not be calculated. Method linear range, MDLs, and MQLs, expressed in ng/L, were estimated from the instrumental ones (see in the) and are presented in Table S2. The MDLs ranged from 0.3 to 2 ng/L, while the MQLs (lowest concentration within the linear range) ranged from 1 to 20 ng/L for most analytes among the different matrices. The exceptions were FEN and FLB, whose instrumental limits were significantly higher due to their low ionization yield. Consequently, higher method limits were obtained for these two compounds.

Precision was assessed in terms of repeatability intra-day (%RSD, n = 5) and reproducibility between days (%RSD, n = 5) at the same concentration level used for recovery evaluation. Repeatability values were below 15%, and reproducibility values were below 20%, regardless of the sample type.

Finally, it was found that the SPE cartridges could be re-used up to 40 times (comparing the results among uses) without significant loss of performance, confirming the robustness of the method and reusability of the core-shell sorbent.

### Analysis of real samples

 Once the SPE-LC–MS/MS method was validated, it was applied to environmental water samples for the determination of the seven acidic and six basic pharmaceuticals. Specifically, three Ebre river water samples, three effluent wastewater samples and three influent wastewater samples collected in the Tarragona region (Spain) were analyzed. Quantification of the target analytes was performed by external calibration, considering preconcentration factors and apparent recoveries for each compound.

Table 2 presents the concentration ranges determined in river water, effluent wastewater, and influent wastewater samples. In river water samples, most compounds were detected at the lowest concentrations among all matrices analyzed. FEN was not detected, while CLO and FLB were found below the MDL and MQL, respectively. On the other hand, NPX was the compound found at highest concentration in one river water sample, reaching a concentration of 834 ng/L. Borrull et al*.* [27] also reported the presence of several pharmaceuticals in Ebre river water, with comparable concentrations for CLO (0.6–45 ng/L), and FEN (not detected). Moreover, higher concentrations of TRI, MTO, and ATE (up to 398, 117, and 92 ng/L, respectively) were reported in their study. Similarly, Gorito et al*.* [28] detected TRI at concentrations below 40 ng/L in the Leça River (Portugal), in accordance with the levels of TRI observed in the present work.

**Table 2:** Concentration ranges (in ng/L) of analytes found in environmental samples (n = 3)

		Ebre river water (ng/L)	Effluent wastewater (ng/L)	Influent wastewater (ng/L)
Basic	ATE	18–28	615–657	2918–3902
	RAN	5–16	5–20	16–41
	TRI	22–52	54–69	422–2846
	MTO	25–51	8–39	67–1410
	VEN	24–59	116–187	1023–2591
	PRO	66–279	22–36	125–2351
Acidic	CLO	< MDL	< MDL–546	< MDL–1092
	NPX	15–834	2077–5258	-
	BEZ	20–251	44–82	348–929
	VAL	10–44	1446–2501	-
	FEN	nd	1834–2225	4590–5088
	FLB	< MQL–324	< MQL–599	< MQL–2094
	DICLO	17–83	378–775	2390–3492

For effluent wastewater samples, NPX and VAL were found at the highest concentrations, ranging from 2077 to 5258 ng/L and from 1446 to 2501 ng/L, respectively. Furthermore, ATE and DICLO were present at lower concentrations than NPX and VAL, although still at relatively elevated levels compared to the remaining compounds, with maximum concentrations of 775 ng/L. Comparable concentration ranges for similar analytes in effluent wastewater were also reported by Salas et al*.* [29], who quantified DICLO at concentrations between 433 and 905 ng/L, in agreement with the present study (378–775 ng/L). However, they reported considerably higher ATE concentrations (1037–2551 ng/L), whereas ATE concentrations in the present study did not exceed 660 ng/L.

As expected, influent wastewater samples presented the highest concentrations for most target compounds (see Fig. 4). The only exception was RAN, whose low concentration remained relatively constant across the different sample types. NPX and VAL could not be quantified in influent wastewater samples, as %R_app_ values could not be calculated due to their high concentrations, similarly to observations reported by Moral et al*.* [14]. In that study [14], comparable influent wastewater concentrations were reported for MTO (up to 1414 ng/L), whereas TRI was found at much higher concentrations (19,137 ng/L in one sample). In contrast, Mostafa et al*.* [30], reported lower influent wastewater concentrations for ATE, MTO, and TRI than those in the present study, ranging from 249 to 325, 236 to 457, and 67 to 204 ng/L, respectively.

## Conclusions

In this study, core-shell polymer microspheres having amphipathic and amphoteric properties have been prepared and deployed successfully as an SPE sorbent for the simultaneous solid-phase extraction of acidic and basic pharmaceutical compounds from environmental water samples. The microspheres are small (3.7 µm) and uniform (C_v_ = 5.5%) and have thin (~ 0.5 µm) hypercrosslinked shells that have very high specific surface area (> 1300 m^2^/g) and are decorated with functional groups which support WAX and WCX analyte retention mechanisms. When applied as sorbent to SPE, loading samples at pH 6 enabled the effective retention of both acidic and basic analytes, with recoveries between 19 and 48% and between 55 and 88%, respectively. Additionally, the inclusion of a methanol-based clean-up step reduced matrix interferences, decreased overall matrix effects, and enhanced selectivity, particularly for basic compounds. Finally, the application of the method to environmental samples confirmed the presence of most of the target compounds in the ng/L concentration level, demonstrating the suitability and applicability of the proposed sorbent for the reliable detection of emerging contaminants in environmental matrices.

## Supplementary Information

Below is the link to the electronic supplementary material.

Supplementary file1 (DOCX 20.1 MB)

## Data Availability

Data are available from the authors by request.
