# Supplementary material for: Hypercrosslinked core-shell polymer microspheres with amphipathic and amphoteric character for the solid-phase extraction of acidic and basic pharmaceuticals from environmental waters
Source: Anal Bioanal Chem. 2026 May 28;418(18):6115–27. doi: 10.1007/s00216-026-06578-z (PMC13541891; doi:10.1007/s00216-026-06578-z)
Supplement: Supplementary file 1 [file 216_2026_6578_MOESM1_ESM.docx]

**Electronic supplementary materials**

Synthesis of core-shell polymer microspheres with amphipathic and amphoteric character (see Figure S1)

**Step 1**. Precipitation polymerisation (PP) of DVB-55 to give polyDVB-55 seed particles

DVB-55 (8.23 g, 63.22 mmol) and AIBN (0.331 g, 2.02 mmol) were dissolved in ACN (400 mL) in a 1 L Nalgene^®^ bottle. The solution was deoxygenated by ultrasonication using an ultrasonic bath for 10 minutes followed by sparging of the solution with N_2_ gas for 15 minutes. The bottle was then sealed under N_2_, placed onto a Stovall low-profile roller (which was housed inside a Stuart Scientific S160D incubator), and rolled about its long axis at a rate of 5-10 rpm. The temperature of the incubator was then ramped from ambient temperature to 60 °C over the course of approximately 2 hours and held at 60 °C for a further 22 hours. Once the polymerisation was complete, the bottle contents were in the form of a milky white suspension of polymer particles. After cooling, a drop of the crude reaction mixture was spotted onto a microscope slide and the particles visualised using optical microscopy. The particles were then isolated by vacuum filtration on a 0.45 μm nylon membrane filter and washed on the filter with successive volumes (100 mL) of ACN and acetone. After drying overnight *in vacuo* (0.01 bar) at 40 °C, the product was isolated as a free-flowing white powder (2.00 g, 23%).

FTIR v̄_max_/cm^-1^: 3022 (aromatic C-H str.), 2933 (aliphatic C-H str.), 837 (1,4-disubstituted aromatic out-of-plane C-H def.), 799 and 712 (1,3-disubstituted aromatic out-of-plane C-H def.) (Fig. S3). SEM microscopy: mean particle diameter = 2.64 µm, C_v_  = 6%.

**Step 2**. Shelling of the polyDVB-55 seed particles by the PP of DVB-80 and VBC in the presence of the seed particles to give polyDVB-55@poly(DVB-80-*co*-VBC)

PolyDVB-55 seed particles (1 g), DVB-80 (1.69 g, 12.98 mmol), VBC (1.31 g, 8.58 mmol), AIBN (0.107 g, 0.65 mmol, 2 mol % relative to total number of polymerisable double bonds) and ACN (200 mL) were charged to a 1 L Nalgene^®^ bottle. The mixture was deoxygenated using an ultrasonic bath for 10 minutes followed by sparging of the bottle contents with N_2_ for a further 15 minutes. The bottle was then sealed under N_2_, placed onto a Stovall low-profile roller (which was housed inside a Stuart Scientific S160D incubator) and rolled about its long axis at a rate of 5-10 rpm. The temperature of the incubator was then ramped from ambient temperature to 60 °C over the course of approximately 2 hours and held at 60 °C for a further 46 hours. Once the polymerisation was complete, the bottle contents were in the form of a milky white suspension of polymer particles. After cooling, a drop of crude reaction mixture was spotted onto a microscope slide and the particles visualised using optical microscopy. The particles were then isolated by vacuum filtration on a 0.45 μm nylon membrane filter and washed on the filter with successive volumes (100 mL) of ACN and acetone. After drying overnight *in vacuo* (0.01 bar) at 40 °C, the product was isolated as a free-flowing white powder (1.84 g, 28 %).

FTIR v̄_max_/cm^-1^: 3031 (aromatic C-H str.), 2934 (aliphatic C-H str.), 1272 (CH_2_-Cl wag), 835 (1,4-disubstituted aromatic out-of-plane C-H def.), 799 and 713 (1,3-disubstituted aromatic out-of-plane C-H def.) (Fig. S4). SEM microscopy: mean particle diameter =3.37 µm, C_v_  = 5%. Langmuir specific surface area = < 5 m^2^/g (Fig. S8).

**Step 3**. Hypercrosslinking of the polyDVB-55@poly(DVB-80-*co*-VBC)

PolyDVB-55@poly(DVB-80-*co*-VBC) microspheres were added to a three-necked, round-bottomed flask together with anhydrous DCE (40 mL). An overhead stirrer and reflux condenser were fitted to the flask, the suspension of microspheres in DCE was sparged with N_2_ gas and the mixture stirred for 1-2 hours at a rate of 50 rpm. Once the microspheres were fully solvated, FeCl_3_ (0.58 g, 3.58 mmol, mass based on 1:1 stoichiometry with CH_2_Cl groups) was added to the reaction vessel. The temperature and stirring rate were increased to 75 °C (from room temperature) and 80 rpm, respectively, for a further ten minutes. After this time, the reaction mixture was dark purple. After cooling, the particles were isolated by vacuum filtration on a 0.45 μm nylon membrane filter and washed on the filter with successive volumes (25 mL) of each of the following solvents: ACN, methanol, 0.01 M NaHCO_3_, distilled water and acetone. After drying overnight *in vacuo* (0.01 bar) at 40 °C, the product was isolated as a free-flowing yellow powder (1.37 g, 91%).

FTIR v̄_max_/cm^-1^: 3027 (aromatic C-H str.), 2928 (aliphatic C-H str.), 1272 (CH_2_-Cl wag), 831 (1,4-disubstituted aromatic out-of-plane C-H def.), 797 and 712 (1,3-disubstituted aromatic out-of-plane C-H def.) (Fig. S5). SEM microscopy: mean particle diameter = 3.76 µm, C_v_  = 5%. Langmuir specific surface area = 858 m^2^/g; specific pore volume = 0.33 cm^3^/g; mean pore diameter = 3.2 nm (Fig. S9).

**Step 4**. Chemical immobilization of sarcosine ethyl ester through a polymer-analogous reaction on pendent chloromethyl groups

Hypercrosslinked polyDVB-55@poly(DVB-80-*co*-VBC) microspheres (1.2 g), sarcosine ethyl ester hydrochloride (1.66 g, 10.80 mmol), tetra-*n*-butylammonium bromide (0.93 g, 2.88 mmol) and sodium hydrogen carbonate (1.99 g, 23.69 mmol) were added to a three-necked round bottom flask together with toluene (40 mL). An overhead stirrer and reflux condenser were fitted to the flask. The flask was immersed in a temperature controllable oil bath and was heated to 80 °C for 24 hours with stirring (80 rpm). After cooling, the particles were isolated on a 0.45 μm nylon membrane filter and washed on the filter with successive volumes (50 mL) of distilled water and acetone. After drying overnight *in vacuo* (0.01 bar) at 40 °C, the product was isolated as a free-flowing pale orange powder (1.13 g, 97 %).

FTIR v̄_max_/cm^-1^: 3022 (aromatic C-H str.), 2939 (aliphatic C-H str.), 1712 (ester C=O str.), 1270 (CH_2_-Cl wag), 1185 (ester C-O str.), 1024 (amine C-N str.), 827 (1,4-disubstituted aromatic out-of-plane C-H def.), 796 and 712 (1,3-disubstituted aromatic out-of-plane C-H def.) (Fig. S6). Optical microscopy: mean particle diameter = 3.69 µm, C_v_ = 5%. Langmuir specific surface area = 796 m^2^/g; specific pore volume = 0.31 cm^3^/g; mean pore diameter = 3.3 nm (Fig. S10).

**Step 5**. Hydrolysis of the ethyl ester groups present in the immobilized sarcosine ethyl ester residues.

Hypercrosslinked polyDVB-55@poly(DVB-80-*co*-VBC) microspheres functionalized with sarcosine ethyl ester (0.76 g) were added to a solution of KOH (4.28 g, 76.4 mmol) in methanol (100 mL) and placed in a Nalgene^®^ bottle. The bottle was placed onto a Stovall low-profile roller (which was housed inside a Stuart Scientific S160D incubator) and rolled about its long axis at a rate of 5-10 rpm. The reaction was then allowed to mix at ambient temperature for 24 hours. After this time, the particles were isolated by vacuum filtration on a 0.45 μm nylon membrane filter and washed on the filter with MeOH (150 mL). After drying overnight *in vacuo* (0.01 bar) at 40 °C, the product was isolated as a free-flowing pale orange powder (0.73 g, 96 %).

FTIR v̄_max_/cm^-1^: 3027 (aromatic C-H str.), 2925 (aliphatic C-H str.), 1610 ((CO_2_)^-^ asymmetric str.), 1520, 1493, 1452 ((CO_2_)^-^ symmetric str.), 1379, 1271 (CH_2_Cl wag), 1174, 1025 (amine C-N str.), 971, 893, 830 (1,4-disubstituted aromatic out-of-plane C-H def.), 796 and 711 (1,3-disubstituted aromatic out-of-plane C-H def.) (Fig. S7). SEM microscopy: mean particle diameter = 3.70 µm, C_v_ = 5.5%. Langmuir specific surface area = 820 m^2^/g; specific pore volume = 0.32 cm^3^/g; mean pore diameter = 3.2 nm (Fig. S11).

Estimation of the Specific Surface Area of the Shells

Radius of core particles = 1.32 µm

Radius of core-shell particles = 1.85 µm

Since the volume of a sphere =$\frac{4}{3}\pi r^{3}$, for a core-shell particle where the radius of the core is 1.32 µm and the radius of the core-shell particle is 1.85 µm then the core contributes 36% of the total volume of the core-shell particle and the shell contributes 64% of the total volume of the core-shell particle.

If one assumes that the core and the shell have the same density and given that the specific surface area of the core-shell material in 820 m^2^/g, then the specific surface area of the shell is 1281 m^2^/g (since 820 x $\frac{1}{0.64}$ = 1281).

However, since the porous shell will have lower density than the non-porous core (in other words, although the shell contributes 64% of the volume it contributes < 64% of the mass), then 1281 m^2^/g is an underestimate of the specific surface area of the shell, hence the specific surface area of the shell will be > 1,300 m^2^/g. This specific surface area value is in line with the specific surface areas of hypercrosslinked polymer microspheres reported elsewhere in the literature (see., e.g., Adv. Mater. (2008) 20, 1298–1302).

**Tables**

Table S1. The 13 pharmaceuticals used as analytes in the SPE study, including information on chemical structures, pK_a_ values, MRM transitions, collision energies and fragmentor voltages (the latter by ESI (+) ionization in MS/MS).

*Bold indicates the quantification transitions

^a^Obtained from PubChem or Drugbank

^b^ESI (-) ionization

Table S2. Estimated method detection limits (MDLs) and linear ranges for the different water samples.

**Figures**

Figure S1: Reaction scheme outlining the synthesis of the core-shell polymer microspheres: **Step 1**. Precipitation polymerisation (PP) of DVB-55 to give polyDVB-55 seed particles; **Step 2**. Shelling of the polyDVB-55 seed particles by the PP of DVB-80 and VBC in the presence of the seed particles to give polyDVB-55@poly(DVB-80-*co*-VBC); **Step 3**. Hypercrosslinking of the poly(DVB-80-*co*-VBC) shells; **Step 4**. Chemical immobilization of sarcosine ethyl ester through a polymer-analogous reaction on pendent chloromethyl groups; **Step 5**. Hydrolysis of the ethyl ester groups present in the immobilized sarcosine ethyl ester residues.

Figure S2a: SEM micrograph of polyDVB-55 seed (core) particles (scale bar = 20 µm).

Figure S2b: SEM micrograph of poly(DVB-55)@poly(DVB-80-co-VBC) core-shell particles (scale bar = 20 µm).

Figure S2c: SEM micrograph of hypercrosslinked poly(DVB-55)@poly(DVB-80-co-VBC) core-shell microspheres (scale bar = 20 µm).

Figure S2d: Optical microscopy image of core-shell polymer microspheres, where the shells are hypercrosslinked and functionalised with sarcosine ethyl ester moieties (product from synthetic Step 4 in Figure S1) (scale bar = 50 µm).

Figure S2e: SEM micrograph of core-shell polymer microspheres, where the shells have amphipathic and amphoteric character arising from hypercrosslinks (amphipathic properties) and immobilised sarcosine moities (amphoteric properties) (product from synthetic Step 5 in Figure S1) (scale bar = 20 µm).

| Wavenumber (cm-1) | Transmittance (%) |
| --- | --- |
| 449.815 | 97.2702 |
| 450.297 | 97.46 |
| 450.779 | 97.6272 |
| 451.261 | 97.7447 |
| 451.743 | 97.8004 |
| 452.226 | 97.7991 |
| 452.708 | 97.7549 |
| 453.19 | 97.6848 |
| 453.672 | 97.6011 |
| 454.154 | 97.5122 |
| 454.636 | 97.4236 |
| 455.118 | 97.3407 |
| 455.6 | 97.2715 |
| 456.082 | 97.2253 |
| 456.565 | 97.2092 |
| 457.047 | 97.2251 |
| 457.529 | 97.2668 |
| 458.011 | 97.3225 |
| 458.493 | 97.3756 |
| 458.975 | 97.4103 |
| 459.457 | 97.4177 |
| 459.939 | 97.3965 |
| 460.421 | 97.3542 |
| 460.904 | 97.3004 |
| 461.386 | 97.243 |
| 461.868 | 97.1831 |
| 462.35 | 97.1164 |
| 462.832 | 97.0353 |
| 463.314 | 96.9331 |
| 463.796 | 96.8077 |
| 464.278 | 96.6614 |
| 464.76 | 96.5008 |
| 465.243 | 96.3358 |
| 465.725 | 96.1773 |
| 466.207 | 96.0354 |
| 466.689 | 95.9179 |
| 467.171 | 95.8307 |
| 467.653 | 95.7771 |
| 468.135 | 95.7598 |
| 468.617 | 95.7797 |
| 469.099 | 95.8361 |
| 469.582 | 95.9261 |
| 470.064 | 96.0443 |
| 470.546 | 96.1842 |
| 471.028 | 96.3397 |
| 471.51 | 96.5049 |
| 471.992 | 96.6751 |
| 472.474 | 96.8436 |
| 472.957 | 97.001 |
| 473.439 | 97.1363 |
| 473.921 | 97.2384 |
| 474.403 | 97.3012 |
| 474.885 | 97.3269 |
| 475.367 | 97.328 |
| 475.849 | 97.3237 |
| 476.331 | 97.3331 |
| 476.813 | 97.3706 |
| 477.296 | 97.4409 |
| 477.778 | 97.5387 |
| 478.26 | 97.651 |
| 478.742 | 97.7609 |
| 479.224 | 97.853 |
| 479.706 | 97.9146 |
| 480.188 | 97.9391 |
| 480.67 | 97.926 |
| 481.152 | 97.8804 |
| 481.635 | 97.8114 |
| 482.117 | 97.7293 |
| 482.599 | 97.6445 |
| 483.081 | 97.564 |
| 483.563 | 97.4906 |
| 484.045 | 97.4234 |
| 484.527 | 97.3583 |
| 485.009 | 97.2899 |
| 485.491 | 97.2144 |
| 485.974 | 97.1313 |
| 486.456 | 97.0449 |
| 486.938 | 96.9636 |
| 487.42 | 96.897 |
| 487.902 | 96.8543 |
| 488.384 | 96.841 |
| 488.866 | 96.8564 |
| 489.348 | 96.8944 |
| 489.831 | 96.9446 |
| 490.313 | 96.9959 |
| 490.795 | 97.0387 |
| 491.277 | 97.0673 |
| 491.759 | 97.0801 |
| 492.241 | 97.0797 |
| 492.723 | 97.0716 |
| 493.205 | 97.062 |
| 493.688 | 97.057 |
| 494.17 | 97.0606 |
| 494.652 | 97.0741 |
| 495.134 | 97.096 |
| 495.616 | 97.1232 |
| 496.098 | 97.152 |
| 496.58 | 97.1801 |
| 497.062 | 97.2077 |
| 497.544 | 97.2381 |
| 498.027 | 97.2763 |
| 498.509 | 97.3274 |
| 498.991 | 97.3929 |
| 499.473 | 97.4699 |
| 499.955 | 97.55 |
| 500.437 | 97.6219 |
| 500.919 | 97.6733 |
| 501.401 | 97.6965 |
| 501.884 | 97.6896 |
| 502.366 | 97.6576 |
| 502.848 | 97.6089 |
| 503.33 | 97.5533 |
| 503.812 | 97.4985 |
| 504.294 | 97.4481 |
| 504.776 | 97.4021 |
| 505.258 | 97.3582 |
| 505.74 | 97.3145 |
| 506.223 | 97.2714 |
| 506.705 | 97.2319 |
| 507.187 | 97.1997 |
| 507.669 | 97.1774 |
| 508.151 | 97.165 |
| 508.633 | 97.1589 |
| 509.115 | 97.1532 |
| 509.597 | 97.1419 |
| 510.079 | 97.1216 |
| 510.562 | 97.0927 |
| 511.044 | 97.0593 |
| 511.526 | 97.0269 |
| 512.008 | 97.0002 |
| 512.49 | 96.9807 |
| 512.972 | 96.9668 |
| 513.454 | 96.9547 |
| 513.936 | 96.9408 |
| 514.418 | 96.9239 |
| 514.901 | 96.9064 |
| 515.383 | 96.8932 |
| 515.865 | 96.8893 |
| 516.347 | 96.8975 |
| 516.829 | 96.9166 |
| 517.311 | 96.9411 |
| 517.793 | 96.9634 |
| 518.275 | 96.9764 |
| 518.758 | 96.9775 |
| 519.24 | 96.9691 |
| 519.722 | 96.9594 |
| 520.204 | 96.9594 |
| 520.686 | 96.9799 |
| 521.168 | 97.0265 |
| 521.65 | 97.0982 |
| 522.132 | 97.1874 |
| 522.615 | 97.2822 |
| 523.097 | 97.369 |
| 523.579 | 97.4358 |
| 524.061 | 97.4753 |
| 524.543 | 97.4852 |
| 525.025 | 97.4675 |
| 525.507 | 97.4273 |
| 525.989 | 97.3702 |
| 526.471 | 97.3011 |
| 526.954 | 97.2233 |
| 527.436 | 97.1393 |
| 527.918 | 97.051 |
| 528.4 | 96.9608 |
| 528.882 | 96.8717 |
| 529.364 | 96.7871 |
| 529.846 | 96.7101 |
| 530.328 | 96.6435 |
| 530.811 | 96.5888 |
| 531.293 | 96.5464 |
| 531.775 | 96.5151 |
| 532.257 | 96.4931 |
| 532.739 | 96.4777 |
| 533.221 | 96.4667 |
| 533.703 | 96.4581 |
| 534.185 | 96.451 |
| 534.667 | 96.4453 |
| 535.149 | 96.4411 |
| 535.632 | 96.4379 |
| 536.114 | 96.4344 |
| 536.596 | 96.4286 |
| 537.078 | 96.4183 |
| 537.56 | 96.4025 |
| 538.042 | 96.3822 |
| 538.524 | 96.3618 |
| 539.006 | 96.3477 |
| 539.489 | 96.3468 |
| 539.971 | 96.3645 |
| 540.453 | 96.402 |
| 540.935 | 96.4559 |
| 541.417 | 96.5184 |
| 541.899 | 96.5792 |
| 542.381 | 96.6297 |
| 542.863 | 96.6647 |
| 543.346 | 96.685 |
| 543.828 | 96.6961 |
| 544.31 | 96.7072 |
| 544.792 | 96.7266 |
| 545.274 | 96.7587 |
| 545.756 | 96.8021 |

Figure S3: FTIR spectrum of polyDVB-55.

| Wavenumber (cm-1) | Transmittance (%) |
| --- | --- |
| 449.815 | 96.0481 |
| 450.297 | 96.0186 |
| 450.779 | 96.0131 |
| 451.261 | 96.0273 |
| 451.743 | 96.0504 |
| 452.226 | 96.068 |
| 452.708 | 96.0666 |
| 453.19 | 96.0353 |
| 453.672 | 95.9701 |
| 454.154 | 95.8743 |
| 454.636 | 95.7594 |
| 455.118 | 95.6424 |
| 455.6 | 95.5432 |
| 456.082 | 95.4789 |
| 456.565 | 95.4593 |
| 457.047 | 95.4857 |
| 457.529 | 95.5506 |
| 458.011 | 95.64 |
| 458.493 | 95.7344 |
| 458.975 | 95.8135 |
| 459.457 | 95.8616 |
| 459.939 | 95.8712 |
| 460.421 | 95.8464 |
| 460.904 | 95.7996 |
| 461.386 | 95.7475 |
| 461.868 | 95.7038 |
| 462.35 | 95.6742 |
| 462.832 | 95.6566 |
| 463.314 | 95.644 |
| 463.796 | 95.6288 |
| 464.278 | 95.6069 |
| 464.76 | 95.5805 |
| 465.243 | 95.5563 |
| 465.725 | 95.544 |
| 466.207 | 95.5495 |
| 466.689 | 95.5721 |
| 467.171 | 95.6018 |
| 467.653 | 95.6231 |
| 468.135 | 95.6193 |
| 468.617 | 95.5775 |
| 469.099 | 95.4945 |
| 469.582 | 95.3786 |
| 470.064 | 95.2489 |
| 470.546 | 95.128 |
| 471.028 | 95.037 |
| 471.51 | 94.9898 |
| 471.992 | 94.9908 |
| 472.474 | 95.0374 |
| 472.957 | 95.1218 |
| 473.439 | 95.2345 |
| 473.921 | 95.3644 |
| 474.403 | 95.4994 |
| 474.885 | 95.6273 |
| 475.367 | 95.735 |
| 475.849 | 95.8106 |
| 476.331 | 95.8461 |
| 476.813 | 95.839 |
| 477.296 | 95.7939 |
| 477.778 | 95.7221 |
| 478.26 | 95.6401 |
| 478.742 | 95.5653 |
| 479.224 | 95.512 |
| 479.706 | 95.4894 |
| 480.188 | 95.5002 |
| 480.67 | 95.5409 |
| 481.152 | 95.6031 |
| 481.635 | 95.6766 |
| 482.117 | 95.7514 |
| 482.599 | 95.8182 |
| 483.081 | 95.8695 |
| 483.563 | 95.8999 |
| 484.045 | 95.9056 |
| 484.527 | 95.8859 |
| 485.009 | 95.8439 |
| 485.491 | 95.7874 |
| 485.974 | 95.7274 |
| 486.456 | 95.6759 |
| 486.938 | 95.6428 |
| 487.42 | 95.6329 |
| 487.902 | 95.6448 |
| 488.384 | 95.6717 |
| 488.866 | 95.7049 |
| 489.348 | 95.7365 |
| 489.831 | 95.7622 |
| 490.313 | 95.7823 |
| 490.795 | 95.8007 |
| 491.277 | 95.8217 |
| 491.759 | 95.8476 |
| 492.241 | 95.8777 |
| 492.723 | 95.9073 |
| 493.205 | 95.9308 |
| 493.688 | 95.943 |
| 494.17 | 95.9411 |
| 494.652 | 95.9259 |
| 495.134 | 95.9002 |
| 495.616 | 95.868 |
| 496.098 | 95.8319 |
| 496.58 | 95.7932 |
| 497.062 | 95.7519 |
| 497.544 | 95.7081 |
| 498.027 | 95.6626 |
| 498.509 | 95.6185 |
| 498.991 | 95.5799 |
| 499.473 | 95.5518 |
| 499.955 | 95.5378 |
| 500.437 | 95.5389 |
| 500.919 | 95.5531 |
| 501.401 | 95.575 |
| 501.884 | 95.5979 |
| 502.366 | 95.6147 |
| 502.848 | 95.6201 |
| 503.33 | 95.6117 |
| 503.812 | 95.591 |
| 504.294 | 95.5629 |
| 504.776 | 95.5359 |
| 505.258 | 95.5199 |
| 505.74 | 95.5223 |
| 506.223 | 95.5474 |
| 506.705 | 95.5934 |
| 507.187 | 95.6533 |
| 507.669 | 95.7159 |
| 508.151 | 95.7685 |
| 508.633 | 95.8006 |
| 509.115 | 95.8065 |
| 509.597 | 95.7873 |
| 510.079 | 95.7502 |
| 510.562 | 95.7062 |
| 511.044 | 95.6659 |
| 511.526 | 95.6355 |
| 512.008 | 95.6153 |
| 512.49 | 95.6001 |
| 512.972 | 95.5823 |
| 513.454 | 95.5555 |
| 513.936 | 95.5178 |
| 514.418 | 95.473 |
| 514.901 | 95.4296 |
| 515.383 | 95.3974 |
| 515.865 | 95.3831 |
| 516.347 | 95.3879 |
| 516.829 | 95.4056 |
| 517.311 | 95.4247 |
| 517.793 | 95.4315 |
| 518.275 | 95.4152 |
| 518.758 | 95.371 |
| 519.24 | 95.3022 |
| 519.722 | 95.2192 |
| 520.204 | 95.1366 |
| 520.686 | 95.0698 |
| 521.168 | 95.0289 |
| 521.65 | 95.0175 |
| 522.132 | 95.0318 |
| 522.615 | 95.0629 |
| 523.097 | 95.0991 |
| 523.579 | 95.129 |
| 524.061 | 95.1449 |
| 524.543 | 95.143 |
| 525.025 | 95.1246 |
| 525.507 | 95.0946 |
| 525.989 | 95.0596 |
| 526.471 | 95.0261 |
| 526.954 | 94.9983 |
| 527.436 | 94.9774 |
| 527.918 | 94.9605 |
| 528.4 | 94.943 |
| 528.882 | 94.9187 |
| 529.364 | 94.8839 |
| 529.846 | 94.8385 |
| 530.328 | 94.7866 |
| 530.811 | 94.7356 |
| 531.293 | 94.6932 |
| 531.775 | 94.6658 |
| 532.257 | 94.6556 |
| 532.739 | 94.6603 |
| 533.221 | 94.6732 |
| 533.703 | 94.6858 |
| 534.185 | 94.69 |
| 534.667 | 94.6805 |
| 535.149 | 94.6555 |
| 535.632 | 94.6175 |
| 536.114 | 94.5721 |
| 536.596 | 94.5256 |
| 537.078 | 94.4839 |
| 537.56 | 94.4514 |
| 538.042 | 94.4302 |
| 538.524 | 94.4205 |
| 539.006 | 94.4209 |
| 539.489 | 94.4286 |
| 539.971 | 94.4402 |
| 540.453 | 94.452 |
| 540.935 | 94.4609 |
| 541.417 | 94.4647 |
| 541.899 | 94.4628 |
| 542.381 | 94.4567 |
| 542.863 | 94.4491 |
| 543.346 | 94.4442 |
| 543.828 | 94.4456 |
| 544.31 | 94.456 |
| 544.792 | 94.4763 |
| 545.274 | 94.5055 |
| 545.756 | 94.541 |

Figure S4: FTIR spectrum of polyDVB-55@poly(DVB-80-*co*-VBC).

| Wavenumber (cm-1) | Transmittance (%) |
| --- | --- |
| 449.815 | 98.0746 |
| 450.297 | 98.1749 |
| 450.779 | 98.2676 |
| 451.261 | 98.3309 |
| 451.743 | 98.3509 |
| 452.226 | 98.3247 |
| 452.708 | 98.2572 |
| 453.19 | 98.1582 |
| 453.672 | 98.0381 |
| 454.154 | 97.9081 |
| 454.636 | 97.7797 |
| 455.118 | 97.6624 |
| 455.6 | 97.5651 |
| 456.082 | 97.4936 |
| 456.565 | 97.4501 |
| 457.047 | 97.4335 |
| 457.529 | 97.4403 |
| 458.011 | 97.4654 |
| 458.493 | 97.5035 |
| 458.975 | 97.5495 |
| 459.457 | 97.5992 |
| 459.939 | 97.6486 |
| 460.421 | 97.6927 |
| 460.904 | 97.7259 |
| 461.386 | 97.7422 |
| 461.868 | 97.7369 |
| 462.35 | 97.7095 |
| 462.832 | 97.6644 |
| 463.314 | 97.6105 |
| 463.796 | 97.5598 |
| 464.278 | 97.5251 |
| 464.76 | 97.5166 |
| 465.243 | 97.5396 |
| 465.725 | 97.5936 |
| 466.207 | 97.6724 |
| 466.689 | 97.7664 |
| 467.171 | 97.8651 |
| 467.653 | 97.9605 |
| 468.135 | 98.0466 |
| 468.617 | 98.1184 |
| 469.099 | 98.172 |
| 469.582 | 98.2026 |
| 470.064 | 98.2065 |
| 470.546 | 98.1826 |
| 471.028 | 98.1329 |
| 471.51 | 98.0632 |
| 471.992 | 97.9808 |
| 472.474 | 97.894 |
| 472.957 | 97.8106 |
| 473.439 | 97.7365 |
| 473.921 | 97.6763 |
| 474.403 | 97.6333 |
| 474.885 | 97.6089 |
| 475.367 | 97.6038 |
| 475.849 | 97.6159 |
| 476.331 | 97.6414 |
| 476.813 | 97.6735 |
| 477.296 | 97.703 |
| 477.778 | 97.7207 |
| 478.26 | 97.7183 |
| 478.742 | 97.6912 |
| 479.224 | 97.6401 |
| 479.706 | 97.5711 |
| 480.188 | 97.4946 |
| 480.67 | 97.4229 |
| 481.152 | 97.3681 |
| 481.635 | 97.3386 |
| 482.117 | 97.3374 |
| 482.599 | 97.3624 |
| 483.081 | 97.4077 |
| 483.563 | 97.4655 |
| 484.045 | 97.5273 |
| 484.527 | 97.587 |
| 485.009 | 97.64 |
| 485.491 | 97.6845 |
| 485.974 | 97.7209 |
| 486.456 | 97.7515 |
| 486.938 | 97.7791 |
| 487.42 | 97.8061 |
| 487.902 | 97.8325 |
| 488.384 | 97.8567 |
| 488.866 | 97.8755 |
| 489.348 | 97.8854 |
| 489.831 | 97.8843 |
| 490.313 | 97.8736 |
| 490.795 | 97.8574 |
| 491.277 | 97.8424 |
| 491.759 | 97.8346 |
| 492.241 | 97.8383 |
| 492.723 | 97.8532 |
| 493.205 | 97.8746 |
| 493.688 | 97.8942 |
| 494.17 | 97.9037 |
| 494.652 | 97.8979 |
| 495.134 | 97.8764 |
| 495.616 | 97.8439 |
| 496.098 | 97.8082 |
| 496.58 | 97.7774 |
| 497.062 | 97.7567 |
| 497.544 | 97.7466 |
| 498.027 | 97.7442 |
| 498.509 | 97.745 |
| 498.991 | 97.7458 |
| 499.473 | 97.747 |
| 499.955 | 97.7522 |
| 500.437 | 97.7671 |
| 500.919 | 97.7968 |
| 501.401 | 97.8425 |
| 501.884 | 97.901 |
| 502.366 | 97.9651 |
| 502.848 | 98.0256 |
| 503.33 | 98.0742 |
| 503.812 | 98.1046 |
| 504.294 | 98.1145 |
| 504.776 | 98.1047 |
| 505.258 | 98.0782 |
| 505.74 | 98.0384 |
| 506.223 | 97.988 |
| 506.705 | 97.9288 |
| 507.187 | 97.8627 |
| 507.669 | 97.7929 |
| 508.151 | 97.7247 |
| 508.633 | 97.6648 |
| 509.115 | 97.6199 |
| 509.597 | 97.5953 |
| 510.079 | 97.5926 |
| 510.562 | 97.6096 |
| 511.044 | 97.641 |
| 511.526 | 97.68 |
| 512.008 | 97.7198 |
| 512.49 | 97.7547 |
| 512.972 | 97.7817 |
| 513.454 | 97.7996 |
| 513.936 | 97.8093 |
| 514.418 | 97.8129 |
| 514.901 | 97.8134 |
| 515.383 | 97.8136 |
| 515.865 | 97.8153 |
| 516.347 | 97.8187 |
| 516.829 | 97.8223 |
| 517.311 | 97.8231 |
| 517.793 | 97.8175 |
| 518.275 | 97.8033 |
| 518.758 | 97.7804 |
| 519.24 | 97.7522 |
| 519.722 | 97.724 |
| 520.204 | 97.702 |
| 520.686 | 97.6904 |
| 521.168 | 97.69 |
| 521.65 | 97.6971 |
| 522.132 | 97.7051 |
| 522.615 | 97.7075 |
| 523.097 | 97.6998 |
| 523.579 | 97.6817 |
| 524.061 | 97.6565 |
| 524.543 | 97.6309 |
| 525.025 | 97.6112 |
| 525.507 | 97.6018 |
| 525.989 | 97.6036 |
| 526.471 | 97.6141 |
| 526.954 | 97.6288 |
| 527.436 | 97.6428 |
| 527.918 | 97.6522 |
| 528.4 | 97.6552 |
| 528.882 | 97.6523 |
| 529.364 | 97.6451 |
| 529.846 | 97.635 |
| 530.328 | 97.6227 |
| 530.811 | 97.6074 |
| 531.293 | 97.5873 |
| 531.775 | 97.5605 |
| 532.257 | 97.5256 |
| 532.739 | 97.4829 |
| 533.221 | 97.4345 |
| 533.703 | 97.3841 |
| 534.185 | 97.3364 |
| 534.667 | 97.2972 |
| 535.149 | 97.2704 |
| 535.632 | 97.2585 |
| 536.114 | 97.2611 |
| 536.596 | 97.2751 |
| 537.078 | 97.2962 |
| 537.56 | 97.32 |
| 538.042 | 97.3438 |
| 538.524 | 97.3673 |
| 539.006 | 97.392 |
| 539.489 | 97.4205 |
| 539.971 | 97.4545 |
| 540.453 | 97.4931 |
| 540.935 | 97.533 |
| 541.417 | 97.5687 |
| 541.899 | 97.5939 |
| 542.381 | 97.6046 |
| 542.863 | 97.5992 |
| 543.346 | 97.5799 |
| 543.828 | 97.5508 |
| 544.31 | 97.5165 |
| 544.792 | 97.4807 |
| 545.274 | 97.4444 |
| 545.756 | 97.407 |

Figure S5: FTIR spectrum of hypercrosslinked polyDVB-55@poly(DVB-80-*co*-VBC).

| Wavenumber (cm-1) | Transmittance (%) |
| --- | --- |
| 449.815 | 96.2474 |
| 450.297 | 96.2098 |
| 450.779 | 96.1345 |
| 451.261 | 96.0416 |
| 451.743 | 95.9546 |
| 452.226 | 95.8907 |
| 452.708 | 95.8579 |
| 453.19 | 95.854 |
| 453.672 | 95.8714 |
| 454.154 | 95.9002 |
| 454.636 | 95.9341 |
| 455.118 | 95.972 |
| 455.6 | 96.0176 |
| 456.082 | 96.0748 |
| 456.565 | 96.1443 |
| 457.047 | 96.2202 |
| 457.529 | 96.29 |
| 458.011 | 96.3395 |
| 458.493 | 96.3558 |
| 458.975 | 96.3338 |
| 459.457 | 96.2804 |
| 459.939 | 96.2138 |
| 460.421 | 96.1578 |
| 460.904 | 96.1322 |
| 461.386 | 96.1461 |
| 461.868 | 96.1939 |
| 462.35 | 96.2606 |
| 462.832 | 96.3265 |
| 463.314 | 96.3751 |
| 463.796 | 96.3974 |
| 464.278 | 96.3905 |
| 464.76 | 96.357 |
| 465.243 | 96.3023 |
| 465.725 | 96.2325 |
| 466.207 | 96.153 |
| 466.689 | 96.0695 |
| 467.171 | 95.9896 |
| 467.653 | 95.922 |
| 468.135 | 95.877 |
| 468.617 | 95.8645 |
| 469.099 | 95.8893 |
| 469.582 | 95.9494 |
| 470.064 | 96.0337 |
| 470.546 | 96.1257 |
| 471.028 | 96.2079 |
| 471.51 | 96.2673 |
| 471.992 | 96.2996 |
| 472.474 | 96.3087 |
| 472.957 | 96.3047 |
| 473.439 | 96.2983 |
| 473.921 | 96.2989 |
| 474.403 | 96.3108 |
| 474.885 | 96.3329 |
| 475.367 | 96.3611 |
| 475.849 | 96.3911 |
| 476.331 | 96.4205 |
| 476.813 | 96.4493 |
| 477.296 | 96.4787 |
| 477.778 | 96.5091 |
| 478.26 | 96.5386 |
| 478.742 | 96.5639 |
| 479.224 | 96.5803 |
| 479.706 | 96.585 |
| 480.188 | 96.5777 |
| 480.67 | 96.5618 |
| 481.152 | 96.5439 |
| 481.635 | 96.5319 |
| 482.117 | 96.5322 |
| 482.599 | 96.5489 |
| 483.081 | 96.5823 |
| 483.563 | 96.6299 |
| 484.045 | 96.686 |
| 484.527 | 96.7437 |
| 485.009 | 96.7943 |
| 485.491 | 96.8287 |
| 485.974 | 96.8391 |
| 486.456 | 96.8198 |
| 486.938 | 96.7703 |
| 487.42 | 96.6953 |
| 487.902 | 96.606 |
| 488.384 | 96.5178 |
| 488.866 | 96.4462 |
| 489.348 | 96.4034 |
| 489.831 | 96.3939 |
| 490.313 | 96.4136 |
| 490.795 | 96.4501 |
| 491.277 | 96.4875 |
| 491.759 | 96.512 |
| 492.241 | 96.5148 |
| 492.723 | 96.4961 |
| 493.205 | 96.4638 |
| 493.688 | 96.4313 |
| 494.17 | 96.4119 |
| 494.652 | 96.4135 |
| 495.134 | 96.437 |
| 495.616 | 96.4757 |
| 496.098 | 96.5189 |
| 496.58 | 96.5554 |
| 497.062 | 96.5784 |
| 497.544 | 96.5872 |
| 498.027 | 96.5868 |
| 498.509 | 96.5853 |
| 498.991 | 96.5898 |
| 499.473 | 96.6042 |
| 499.955 | 96.6271 |
| 500.437 | 96.6529 |
| 500.919 | 96.6732 |
| 501.401 | 96.6802 |
| 501.884 | 96.6692 |
| 502.366 | 96.6392 |
| 502.848 | 96.5936 |
| 503.33 | 96.5391 |
| 503.812 | 96.4852 |
| 504.294 | 96.442 |
| 504.776 | 96.4185 |
| 505.258 | 96.4198 |
| 505.74 | 96.4454 |
| 506.223 | 96.4883 |
| 506.705 | 96.5359 |
| 507.187 | 96.5739 |
| 507.669 | 96.5889 |
| 508.151 | 96.5722 |
| 508.633 | 96.5239 |
| 509.115 | 96.4531 |
| 509.597 | 96.3767 |
| 510.079 | 96.3128 |
| 510.562 | 96.2777 |
| 511.044 | 96.2789 |
| 511.526 | 96.3136 |
| 512.008 | 96.3698 |
| 512.49 | 96.4312 |
| 512.972 | 96.4828 |
| 513.454 | 96.515 |
| 513.936 | 96.5259 |
| 514.418 | 96.5205 |
| 514.901 | 96.5078 |
| 515.383 | 96.4967 |
| 515.865 | 96.4929 |
| 516.347 | 96.499 |
| 516.829 | 96.5138 |
| 517.311 | 96.5352 |
| 517.793 | 96.5606 |
| 518.275 | 96.5884 |
| 518.758 | 96.6165 |
| 519.24 | 96.6415 |
| 519.722 | 96.6583 |
| 520.204 | 96.6612 |
| 520.686 | 96.6446 |
| 521.168 | 96.6074 |
| 521.65 | 96.5524 |
| 522.132 | 96.4873 |
| 522.615 | 96.4222 |
| 523.097 | 96.3667 |
| 523.579 | 96.3285 |
| 524.061 | 96.3105 |
| 524.543 | 96.3115 |
| 525.025 | 96.3274 |
| 525.507 | 96.3531 |
| 525.989 | 96.3833 |
| 526.471 | 96.4133 |
| 526.954 | 96.4391 |
| 527.436 | 96.4566 |
| 527.918 | 96.4622 |
| 528.4 | 96.4543 |
| 528.882 | 96.433 |
| 529.364 | 96.4021 |
| 529.846 | 96.3671 |
| 530.328 | 96.3344 |
| 530.811 | 96.3086 |
| 531.293 | 96.2918 |
| 531.775 | 96.2828 |
| 532.257 | 96.2787 |
| 532.739 | 96.277 |
| 533.221 | 96.2775 |
| 533.703 | 96.282 |
| 534.185 | 96.2944 |
| 534.667 | 96.3174 |
| 535.149 | 96.3507 |
| 535.632 | 96.3901 |
| 536.114 | 96.4275 |
| 536.596 | 96.4538 |
| 537.078 | 96.463 |
| 537.56 | 96.4537 |
| 538.042 | 96.4306 |
| 538.524 | 96.4032 |
| 539.006 | 96.383 |
| 539.489 | 96.3787 |
| 539.971 | 96.3943 |
| 540.453 | 96.4279 |
| 540.935 | 96.4724 |
| 541.417 | 96.518 |
| 541.899 | 96.555 |
| 542.381 | 96.5758 |
| 542.863 | 96.5762 |
| 543.346 | 96.5554 |
| 543.828 | 96.5153 |
| 544.31 | 96.4604 |
| 544.792 | 96.3967 |
| 545.274 | 96.3305 |
| 545.756 | 96.2688 |

Figure S6: FTIR spectrum of core-shell polymer microspheres with immobilized sarcosine ethyl ester.

Figure S7: FTIR spectrum of core-shell polymer microspheres with immobilized sarcosine.

Quantity adsorbed (mmol/g)

| Relative pressure (P/P0) | Adsorption |
| --- | --- |
| 0.0105622 | 0.0203091 |
| 0.0404218 | 0.0270337 |
| 0.0696454 | 0.0303576 |
| 0.0860978 | 0.0320303 |
| 0.11144 | 0.0334958 |
| 0.136748 | 0.0346744 |
| 0.162001 | 0.0357578 |
| 0.187322 | 0.0366923 |
| 0.212611 | 0.0372715 |
| 0.237845 | 0.0378785 |
| 0.263204 | 0.0384774 |
| 0.288319 | 0.0388536 |
| 0.313705 | 0.0390568 |
| 0.339084 | 0.03945 |
| 0.364229 | 0.0395544 |
| 0.409047 | 0.039557 |
| 0.434275 | 0.0396066 |
| 0.440038 | 0.0399118 |
| 0.465373 | 0.0399896 |
| 0.510046 | 0.0400021 |
| 0.535326 | 0.040126 |
| 0.541368 | 0.0408683 |
| 0.566479 | 0.0410104 |
| 0.611138 | 0.0410568 |
| 0.636416 | 0.0415655 |
| 0.642358 | 0.0424817 |
| 0.667725 | 0.0428117 |
| 0.712148 | 0.0436505 |
| 0.718211 | 0.0444449 |
| 0.743451 | 0.0456198 |
| 0.787821 | 0.0477731 |
| 0.79403 | 0.0490353 |
| 0.819539 | 0.0511847 |
| 0.863458 | 0.0561995 |
| 0.870101 | 0.0581825 |
| 0.895568 | 0.0632421 |
| 0.920189 | 0.0704603 |
| 0.945789 | 0.082349 |
| 0.970799 | 0.103542 |
| 0.990371 | 0.145623 |
| 0.996187 | 0.1895 |

| Relative pressure (P/P0) | Desorption |
| --- | --- |
| 0.996187 | 0.18955 |
| 0.957222 | 0.097429 |
| 0.92437 | 0.0764119 |
| 0.895947 | 0.0675072 |
| 0.868142 | 0.0620706 |
| 0.840682 | 0.0582443 |
| 0.813125 | 0.0555739 |
| 0.785644 | 0.0533429 |
| 0.777204 | 0.0527999 |
| 0.74976 | 0.0515665 |
| 0.7223 | 0.0506298 |
| 0.694889 | 0.0500968 |
| 0.667469 | 0.0492674 |
| 0.639998 | 0.0489302 |
| 0.593277 | 0.0485623 |
| 0.565741 | 0.0483593 |
| 0.557737 | 0.0481919 |
| 0.530225 | 0.0482787 |
| 0.483366 | 0.0482368 |
| 0.474531 | 0.0479786 |
| 0.447058 | 0.0480032 |
| 0.400082 | 0.0479863 |
| 0.392286 | 0.047871 |
| 0.364879 | 0.0480066 |
| 0.317791 | 0.0481135 |
| 0.290338 | 0.0478623 |
| 0.282596 | 0.0477439 |
| 0.255289 | 0.047497 |
| 0.227772 | 0.0472971 |
| 0.200553 | 0.0467974 |

Figure S8: BET isotherm of polyDVB-55@poly(DVB-80-*co*-VBC).

Quantity adsorbed (mmol/g)

| Relative pressure (P/P0) | Adsorption |
| --- | --- |
| 0.0100165 | 6.50319 |
| 0.0354211 | 7.26796 |
| 0.0601004 | 7.58035 |
| 0.0873412 | 7.80285 |
| 0.114961 | 7.96633 |
| 0.141132 | 8.09234 |
| 0.168588 | 8.20067 |
| 0.193999 | 8.28981 |
| 0.219089 | 8.36718 |
| 0.243132 | 8.43444 |
| 0.268111 | 8.49677 |
| 0.292728 | 8.55388 |
| 0.316378 | 8.60495 |
| 0.340718 | 8.65401 |
| 0.365145 | 8.7 |
| 0.389019 | 8.74239 |
| 0.415465 | 8.78746 |
| 0.440741 | 8.8273 |
| 0.465991 | 8.86655 |
| 0.491095 | 8.90309 |
| 0.516231 | 8.93812 |
| 0.541401 | 8.97304 |
| 0.566876 | 9.00537 |
| 0.59152 | 9.03637 |
| 0.616995 | 9.06572 |
| 0.641959 | 9.09584 |
| 0.66769 | 9.1253 |
| 0.692672 | 9.15437 |
| 0.718204 | 9.18199 |
| 0.742961 | 9.21002 |
| 0.768942 | 9.23524 |
| 0.79338 | 9.26141 |
| 0.819334 | 9.28846 |
| 0.844273 | 9.31559 |
| 0.869937 | 9.34181 |
| 0.894649 | 9.37076 |
| 0.920501 | 9.40107 |
| 0.945257 | 9.43568 |
| 0.970336 | 9.48461 |
| 0.989588 | 9.56274 |
| 0.994906 | 9.63193 |

| Relative pressure (P/P0) | Desorption |
| --- | --- |
| 0.994906 | 9.63193 |
| 0.956438 | 9.52111 |
| 0.924234 | 9.48265 |
| 0.89586 | 9.45769 |
| 0.886084 | 9.44856 |
| 0.857567 | 9.4303 |
| 0.831385 | 9.4133 |
| 0.803382 | 9.39542 |
| 0.775989 | 9.37789 |
| 0.748542 | 9.3608 |
| 0.721298 | 9.34245 |
| 0.693651 | 9.32422 |
| 0.66663 | 9.30454 |
| 0.638711 | 9.28413 |
| 0.611835 | 9.26344 |
| 0.584049 | 9.24178 |
| 0.556879 | 9.218 |
| 0.529374 | 9.1932 |
| 0.50254 | 9.1668 |
| 0.474898 | 9.13806 |
| 0.447566 | 9.10725 |
| 0.42004 | 9.07431 |
| 0.392944 | 9.03893 |
| 0.36526 | 8.99966 |
| 0.338188 | 8.95745 |
| 0.309814 | 8.90846 |
| 0.281987 | 8.85523 |
| 0.254555 | 8.79713 |
| 0.227676 | 8.73126 |
| 0.19969 | 8.65467 |

Figure S9: BET isotherm of hypercrosslinked polyDVB-55@poly(DVB-80-*co*-VBC).

Quantity adsorbed (mmol/g)

| Relative pressure (P/P0) | Adsorption |
| --- | --- |
| 0.00975516 | 6.04525 |
| 0.0363515 | 6.76408 |
| 0.0605715 | 7.04649 |
| 0.0864674 | 7.23752 |
| 0.114675 | 7.39408 |
| 0.141627 | 7.51291 |
| 0.168996 | 7.61446 |
| 0.194918 | 7.69698 |
| 0.219207 | 7.7651 |
| 0.242886 | 7.82697 |
| 0.267102 | 7.88322 |
| 0.29173 | 7.93657 |
| 0.316592 | 7.98586 |
| 0.34185 | 8.03129 |
| 0.364119 | 8.07155 |
| 0.39008 | 8.11484 |
| 0.415077 | 8.15285 |
| 0.439734 | 8.19609 |
| 0.466168 | 8.22869 |
| 0.489128 | 8.25766 |
| 0.515549 | 8.28923 |
| 0.540658 | 8.31862 |
| 0.565829 | 8.34893 |
| 0.59139 | 8.37718 |
| 0.616336 | 8.40411 |
| 0.641437 | 8.43497 |
| 0.667718 | 8.46006 |
| 0.691542 | 8.48396 |
| 0.717689 | 8.5094 |
| 0.742745 | 8.53315 |
| 0.76781 | 8.55903 |
| 0.793293 | 8.58521 |
| 0.818631 | 8.60891 |
| 0.843391 | 8.633 |
| 0.868925 | 8.65847 |
| 0.894017 | 8.6856 |
| 0.919335 | 8.71568 |
| 0.944354 | 8.7532 |
| 0.969202 | 8.80987 |
| 0.988443 | 8.90117 |
| 0.994835 | 8.9846 |

| Relative pressure (P/P0) | Desorption |
| --- | --- |
| 0.994835 | 8.9846 |
| 0.957814 | 8.86228 |
| 0.925499 | 8.80385 |
| 0.895822 | 8.7749 |
| 0.867819 | 8.75357 |
| 0.858598 | 8.74607 |
| 0.830167 | 8.73011 |
| 0.803949 | 8.71472 |
| 0.776051 | 8.699 |
| 0.748611 | 8.68309 |
| 0.72104 | 8.667 |
| 0.693827 | 8.65005 |
| 0.666267 | 8.63296 |
| 0.639058 | 8.61477 |
| 0.611487 | 8.59536 |
| 0.584173 | 8.57491 |
| 0.556666 | 8.55408 |
| 0.529356 | 8.53216 |
| 0.501979 | 8.5079 |
| 0.474455 | 8.48158 |
| 0.447051 | 8.45353 |
| 0.419599 | 8.42373 |
| 0.39223 | 8.39208 |
| 0.364997 | 8.3564 |
| 0.337402 | 8.31763 |
| 0.310044 | 8.27523 |
| 0.282611 | 8.22818 |
| 0.255161 | 8.17567 |
| 0.227972 | 8.11655 |
| 0.200342 | 8.04816 |

Figure S10: BET isotherm of core-shell polymer microspheres with immobilized sarcosine ethyl ester.

Quantity Adsorbed (mmol/g)

| Relative Pressure (P/P0) | Adsorption |
| --- | --- |
| 0.00954542 | 6.22351 |
| 0.0340937 | 6.94474 |
| 0.0593315 | 7.25915 |
| 0.0866409 | 7.4666 |
| 0.114722 | 7.62897 |
| 0.141649 | 7.75173 |
| 0.169358 | 7.85308 |
| 0.194785 | 7.93837 |
| 0.218762 | 8.00742 |
| 0.24278 | 8.07049 |
| 0.266373 | 8.12657 |
| 0.291319 | 8.18092 |
| 0.315667 | 8.23131 |
| 0.341279 | 8.27839 |
| 0.363399 | 8.31891 |
| 0.389176 | 8.36251 |
| 0.414176 | 8.40068 |
| 0.43916 | 8.43749 |
| 0.464712 | 8.47185 |
| 0.489547 | 8.50661 |
| 0.515427 | 8.53812 |
| 0.539743 | 8.57109 |
| 0.566048 | 8.60094 |
| 0.590376 | 8.62782 |
| 0.616074 | 8.65447 |
| 0.640473 | 8.68891 |
| 0.668506 | 8.71448 |
| 0.691109 | 8.73771 |
| 0.717693 | 8.76308 |
| 0.742735 | 8.78544 |
| 0.767553 | 8.81029 |
| 0.793536 | 8.83336 |
| 0.818195 | 8.85662 |
| 0.843888 | 8.88096 |
| 0.868782 | 8.90807 |
| 0.894596 | 8.93447 |
| 0.918913 | 8.96561 |
| 0.94514 | 9.00085 |
| 0.969118 | 9.04894 |
| 0.988821 | 9.12675 |
| 0.994802 | 9.19538 |

| Relative Pressure (P/P0) | Desorption |
| --- | --- |
| 0.994802 | 9.19538 |
| 0.9567 | 9.08854 |
| 0.924794 | 9.0453 |
| 0.896105 | 9.01942 |
| 0.868231 | 8.99925 |
| 0.858507 | 8.99122 |
| 0.829942 | 8.97567 |
| 0.803984 | 8.96 |
| 0.775692 | 8.9439 |
| 0.748679 | 8.92773 |
| 0.721085 | 8.91114 |
| 0.693811 | 8.89411 |
| 0.666254 | 8.87674 |
| 0.638934 | 8.85774 |
| 0.611329 | 8.83866 |
| 0.584154 | 8.81841 |
| 0.556609 | 8.79661 |
| 0.529313 | 8.77357 |
| 0.50185 | 8.74882 |
| 0.474446 | 8.72228 |
| 0.447012 | 8.69357 |
| 0.419575 | 8.66326 |
| 0.392246 | 8.63004 |
| 0.364702 | 8.59421 |
| 0.3375 | 8.55509 |
| 0.310212 | 8.51173 |
| 0.282739 | 8.46339 |
| 0.255523 | 8.41012 |
| 0.227911 | 8.34926 |
| 0.200992 | 8.28198 |

Figure S11: BET isotherm of core-shell polymer microspheres with immobilized sarcosine.

Figure S12. Effect of sample volume on recoveries of the different analytes.
